# *Plasmodium vivax* and *Plasmodium falciparum* infections in the Republic of Djibouti: evaluation of their prevalence and potential determinants

**DOI:** 10.1186/1475-2875-11-395

**Published:** 2012-11-28

**Authors:** Bouh Abdi Khaireh, Sébastien Briolant, Aurélie Pascual, Madjid Mokrane, Vanessa Machault, Christelle Travaillé, Mohamed Abdi Khaireh, Ismail Hassan Farah, Habib Moussa Ali, Abdul-Ilah Ahmed Abdi, Souleiman Nour Ayeh, Houssein Youssouf Darar, Lénaïck Ollivier, Mohamed Killeh Waiss, Hervé Bogreau, Christophe Rogier, Bruno Pradines

**Affiliations:** 1 Unité de Parasitologie, Département d’Infectiologie de Terrain, Institut de Recherche Biomédicale des Armées, Marseille, France; 2 Aix Marseille Université, Unité de Recherche sur les Maladies Infectieuses et Tropicales Emergentes, UM 63, CNRS 7278, IRD 198, Inserm 1095, Marseille, France; 3 Service de Santé des Forces Armées Djiboutiennes, Djibouti, République de Djibouti; 4 Service des Maladies Infectieuses et Tropicales, Hôpital Général Peltier, Djibouti, République de Djibouti; 5 Institut de Recherche Médicinale, Centre d’Etude et de Recherche de Djibouti, Djibouti, République de Djibouti; 6 Observatoire Midi-Pyrénées, Laboratoire d’Aérologie, Centre National de le Recherche Scientifique, Université Paul Sabatier, Toulouse, France; 7 Centre National d’Etudes Spatiales, Service Applications et Valorisation, Toulouse, France; 8 Institut National de Santé Publique de Djibouti, Ministère de la Santé, Djibouti, République de Djibouti; 9 Bureau Expertise des risques sanitaires, Sous-direction Action Scientifique et Technique, Direction Centrale du Service de Santé des Armées, Paris, France; 10 Faculté des Sciences, Université de Djibouti, Djibouti, République de Djibouti; 11 Institut Pasteur de Madagascar, Antananarivo, Madagascar

**Keywords:** Malaria, *Plasmodium falciparum*, *Plasmodium vivax*, Seroprevalence, Serological marker, Djibouti

## Abstract

**Background:**

Formerly known as a hypoendemic malaria country, the Republic of Djibouti declared the goal of pre-eliminating malaria in 2006. The aim of the present study was to evaluate the prevalence of *Plasmodium falciparum*, *Plasmodium vivax* and mixed infections in the Djiboutian population by using serological tools and to identify potential determinants of the disease and hotspots of malaria transmission within the country.

**Methods:**

The prevalence of *P*. *falciparum* and *P*. *vivax* within the districts of the capital city and the rest of the Republic of Djibouti were assessed using 13 and 2 serological markers, respectively. The relationship between the immune humeral response to *P*. *falciparum* and *P*. *vivax* and variables such as age, gender, wealth status, urbanism, educational level, distance to rivers/lakes, living area, having fever in the last month, and staying in a malaria-endemic country more than one year was estimated and analysed by questionnaires administered to 1910 Djiboutians. Multivariate ordinal logistic regression models of the immune humeral response were obtained for *P*. *falciparum* and *P*. *vivax*.

**Results:**

The *P*. *falciparum* and *P*. *vivax* seroprevalence rates were 31.5%, CI95% [29.4-33.7] and 17.5%, CI95% [15.8-19.3], respectively. Protective effects against *P*. *falciparum* and *P*. *vivax* were female gender, educational level, and never having visited a malaria-endemic area for more than one year. For *P*. *falciparum* only, a protective effect was observed for not having a fever in the last month, living more than 1.5 km away from lakes and rivers, and younger ages.

**Conclusions:**

This is the first study that assessed the seroprevalence of *P*. *vivax* in the Republic of Djibouti. It is necessary to improve knowledge of this pathogen in order to create an effective elimination programme. As supported by recent observations on the subject, the Republic of Djibouti has probably demonstrated a real decrease in the transmission of *P*. *falciparum* in the past seven years, which should encourage authorities to improve efforts toward elimination.

## Background

According to the World Health Organization (WHO), approximately 3.3 billion people, nearly half of the world population, are at risk of malaria. Each year, approximately 250 million people contract the disease, and nearly one million people die. The inhabitants of the poorest countries are the most vulnerable. More than one in five infant deaths (20%) occurring in Africa are due to malaria [1]. However, the number of *Plasmodium falciparum* malaria cases is declining, even in Africa. Given this situation, in the late 1990s, WHO proposed a goal of controlling the disease and achieving elimination by 2015 in areas of low transmission. Policies, international and national initiatives have proliferated to help the neediest.

Based on the results of scientific research in all areas of malaria control and because of greater knowledge of the disease and its medical and social consequences, the proposed strategy is organized into two main phases: control and disposal [2]. Among the actions undertaken on a large scale, it should be noted that the availability and distribution of ITNs and ACT, vector control through IRS, active detection of new breeding sites and their systematic destruction represent a link essential to the success of disease control before disposal is considered [3].

According to the Roll Back Malaria project, malaria primarily concerns 109 countries, but 35 countries account for 98% of malaria deaths worldwide. Only five of these countries (Nigeria, Democratic Republic of Congo, Uganda, Ethiopia and Tanzania) represent 50% of deaths and 47% of malaria cases [4]. Among these countries, Ethiopia and Uganda share an economic community, bringing together 340 million people who are free to move to the Republic of Djibouti [5].

Formerly known to be a malaria meso- to hypoendemic country with an unstable malaria transmission profile [6-8], this country of 818,159 inhabitants declared a goal of malaria pre-elimination in 2006 [9]. Micro-epidemics can occur in the presence of favourable set of conditions, such as unusual rainfall (the last major outbreak occurred in 1999) [10,11]. Over the last 14 years, the transmission and the malaria cases number remained low. As a result, foreign armies present in the Republic of Djibouti have recently considered stopping their malaria chemoprophylaxis [12], as the French army did last year.

Djibouti has recently demonstrated its eligibility for the pre-elimination goal according to technical feasibility, i.e., the baseline domestic malaria transmission combined with the importation-related transmission and operational feasibility, which takes into consideration the country government status, health status and information on populations at risk [13]. These observations are in agreement with parasite genetic diversity studies [10,11], and one recent work has reported a low transmission level [14].

According to WHO, malaria control requires, at a national level, the expertise and development of databases containing information about the parasites found locally and information about changes in transmission levels and the status of resistance to anti-malarials [15]. Control in the short- and medium-term is possible by developing constantly improved detection and observation tools and early care and adequate diagnoses in risk populations [15].

Therefore, serological tools are widely used to assess the transmission level and thus the prevalence of *Plasmodium falciparum* and *Plasmodium vivax* in human populations and to assess epidemiological facts of the past and present [16,17]. One recent work in Somaliland (the nearest neighbouring country to Djibouti with regular movements of the population in both directions) has used serological tools to assess the prevalence of *P*. *falciparum* and *P*. *vivax*[18-20].

Starting from this observation, it was necessary to perform a similar survey in the Republic of Djibouti. Because most of the previous studies have primarily concerned *P*. *falciparum* malaria, it was also necessary to study *P*. *vivax* malaria and mixed infections to gather enough information for elimination [3].

The aim of the present study was to evaluate the prevalence rate of *P*. *falciparum*, *P*. *vivax* and mixed infections in the Djiboutian population by using serological tools and to identify potential determinants of hot spots of malaria infection and transmission within the country.

## Methods

### Sera samples

The prevalence rate of *P*. *falciparum* and *P*. *vivax* infections among adults aged 15–54 years living in the Republic of Djibouti was estimated using a anonymous non-correlated cluster sampling method between the 24^th^ and the 31^th^ of March 2002. In brief, 30 clusters were investigated in the city of Djibouti, and 25 clusters in the other districts of the country. The clusters were randomly selected proportionally to the population size according to the list of quarters used by the National Direction of Statistics in the city of Djibouti and the list of the towns used by the expanded programme of immunization in the other districts. In each selected site, a starting household was randomly selected, and the next nearest households were investigated until a total of 44 resident adults per cluster in the city of Djibouti or 35 resident adults per cluster in the other districts were obtained. A total of 1,910 blood samples were collected anonymously in accordance with the recommendations of the Djiboutian Ministry of Health, who gave the ethics clearance for the present study. Blood samples were stored at 4 °C until separation of plasma by centrifugation (less than 24 hours after collection) and freezing. Thirty sera samples from French adults who had never been to malaria-endemic countries were used as unexposed negative controls. For the seropositivity threshold, the means and standard deviations (SDs) of the antibody intensity of the negative control group for all antigens were estimated. The lower limit of positivity for each antibody was taken as the mean + 3 SD of the negative control group values and corresponded to a mean fluorescence intensity (MFI) of 1,000. For *P*. *falciparum*, a sample was considered to be positive if the reactivity to at least two different plasmodial antigens was > 1,000 MFI. For *P*. *vivax*, a sample was considered to be positive if the reactivity to PvMSP1-19 or to PvMSP1-42 was > 1,000 MFI.

### Peptides and proteins

Eleven Peptides (Lsa1-41, Lsa1-J, Lsa3-NR2, Glurp, GlurpP3, Salsa1, Salsa2, Trap1, Starp-R, CS (NANP) and SR11.1) were synthesized with an added N-terminal cysteine residue and covalently coupled with BSA (bovine serum albumin, Sigma-Aldrich, St. Louis, USA) by Genpep (Ales, France) and stored in aliquots at −20 °C. *P*. *falciparum* merozoite surface protein 1–19 (MSP1_19_) and apical membrane antigen 1 (AMA1) were obtained using procedures previously described [14]. For *P*. *vivax*, MSP1_19_ proteins were produced in a baculovirus [21] and MSP1_42_ as described elsewhere [19].

### Bead-based assay

Peptides and proteins were coupled to beads as described by Ambrosino *et al*. [22], and an optimal concentration of 0.3 nmol was used for each antigen. Furthermore, BSA coated beads were included as a background control. Ag-coated beads were resuspended by vortexing and sonication for 5 minutes and were diluted in equal volumes of PBS and MFIA (Multiplexed Fluorescence ImmunoAssay) diluent (Charles River Laboratories Inc, MA, USA) to a final concentration of 80 beads/μl per peptide. The 1.2-μm filter-bottom 96-well microtiterplates (MSBVS 1210, Millipore, MA, USA) were rewetted with washing buffer (0.15% Tween 20 in PBS 7.4) using a vacuum manifold (Millipore). Fifty microliters of beads and sera (diluted 1:100 in equal volumes of PBS and MFIA diluents) were added to each well. Plates were incubated at room temperature in the dark for 1 h with shaking at 600 rpm. After incubation, plates were washed eight times with 200 μl of washing buffer, then 100 μl of the secondary Ab (R-phycoerythrin F(ab’)2 fragment of goat anti-human IgG, (Interchim, Montluçon, France), diluted 1:500, was added to each well. After 30 min of incubation in the dark at room temperature with shaking, plates were washed as described previously. Beads were resuspended in 100 μl of a solution of 5% BSA-PBS, pH 7.4 and analysed on Luminex system. The system was set to read a minimum of 100 beads per spectral address, and the results were expressed as MFI.

### Data collection

During the cross-sectional study between the 24^th^ and the 31^th^ March 2002, self-administered questionnaires containing several items were filled out by Djiboutian inhabitants and validated by a member of the research team. Different types of independent variables were collected: the living area (the city of Djibouti or the rest of the country), the type of living area (urban or rural), having stayed in a malaria-endemic country for more than one year (yes or no), having had a fever during the last month before the study (yes or no), the utilization of bed nets (often to always and rarely to never), gender (male or female), schooling status (schooled or never schooled), educational level (never schooled, primary school, secondary school, high school or university), wealth (poor = less than 65,000 Djiboutian Francs per home and rich = more than 65,000 Djiboutian Francs per home), and age.

A Geographic Information System was built in ArcGIS 9.2 (Environmental Research Systems Institute, Redlands, CA). The layers were added as follows: i) map of inland water in Djibouti and the neighbouring countries (data from Digital Chart of the World, accessed through DIVA-GIS [23] and ii) 60 points corresponding to the sampling locations. At every point, the Euclidian distance to the first pixel of water was computed. This enabled the creation of a geographical independent variable, the distance of a cluster to a river or a lake (≤ 1.5 Km or > 1.5 Km).

### Statistical methods

Data were recorded using Excel and were checked for consistency before statistical analysis using R software (version 2.10.1) or STATA software (version 11). The seropositivity to *P*. *falciparum* antigens, *P*. *vivax* antigens or both of them (mixed infection) were analysed as a dependant variable according to individual and cluster characteristics using a random effect mixed logistic regression model. The model was designed to take into account the intracluster correlations that could exist due to the sampling design (cluster effect as random effect). The logistic model was also adjusted using a generalized estimating equations (GEE) approach. Random effect and GEE regression models allow the estimation of cluster-specific and population-averaged effects, respectively [24]. First, a descriptive analysis of the independent variables was performed. A bivariate analysis was then conducted by entering each independent variable in a logistic regression model, and all the results were presented in Additional files 1, 2, 3, 4, 5 and 6. Variables were retained for the multivariate analysis if their effect had a p-value less than 0.25 [25]. A backward stepwise selection procedure was applied to retain the significant (p < 0.05) independent variables and their interactions in the final model. The statistical quality of the final model was assessed by looking at the adequacy between observed and predicted prevalence rates.

As the immune response to several *P*. *falciparum* or *P*. *vivax* antigens could be quantified by MFI, another ordinal dependant variable in four classes was created: the level of immune response to *P*. *falciparum* antigens (L0 = seronegativity MFI < 1000, L1 = 1000 ≤ MFI < 3800, L2 = 3800 ≤ MFI < 8000, and L3 = MFI ≥ 8000) and *P*. *vivax* antigens (L0 = seronegativity MFI < 1000, L1 = 1000 ≤ MFI < 2000, L2 = 2000 ≤ MFI < 10000 and L3 = MFI ≥ 10000). The same type of analysis was applied to this dependent variable as described above for the seropositivity status, using the “svy” command of STATA (i.e., using the linearized variance estimator based on a first-order Taylor series linear approximation) to take into account the cluster effect.

The results of the bivariate Bayesian ordinal multinomial regression analysis are presented in Additional files 7 and 8.

## Results

### *Plasmodium falciparum* seroprevalence

The serological analysis showed that 25.90% of sera were positive for at least two of the following 11 *P*. *falciparum* peptides: Lsa1-41, Lsa1-J, Lsa3-NR2, Glurp, GlurpP3, Salsa1, Salsa2, Trap1, Starp-R, CS (NANP) and SR11.1. The proportion of sera that were positive for both and at least one of the two recombinant *falciparum* antigens, i.e., PfMSP1 and PfAMA1, were 13.24% and 29.98%, respectively. By taking into account the immune humeral response to at least two different peptides or recombinant proteins of the 13 *P*. *falciparum* antigens used in this study, the seropositivity rate to *P*. *falciparum* infection was 31.5% (602/1910 Djiboutian people), CI95% (29.4-33.7).

Considering the potential determinants of the *P*. *falciparum* malaria infection, according to the results of the multivariate logistic regression analysis (Table 1), some factors were significantly and independently statistically associated with a lower risk of being seropositive for *P*. *falciparum*:

– The gender (female gender).

– The educational level (primary school, secondary school and higher levels).

– Never having visited a malaria-endemic area for more than one year.

– Not having had a fever during the last month before the study.

**Table 1:** **Multivariate logistic regression analysis of *P*. *falciparum *infection’s seroprevalence**

	**N**	**P**	**% (95%CI)**	**RE**		
				**cOR (CI95%)**	**aOR (CI95%)**	**p-value**
**Gender**						
Male	742	256	34.5 (31.1-38.0)	1	1	0.0008
Female	1168	346	29.6 (27.0-32.3)	0.78 (0.63-0.96)	0.68 (0.55 - 0.85)	
**Educational level**						
No school	1244	448	36.0 (33.3-38.6)	1	1	0.033
Primary	410	106	25.9 (21.7-30.4)	0.65 (0.50-0.84)	0.66(0.50 - 0.87)	1.6x10^-5^
Secondary, high school or University	256	48	18.8 (14.2-24.1)	0.45 (0.32-0.64)	0.45 (0.31 - 0.64)	
**Staying in a malaria endemic country more than one year**						
Yes	167	70	41.9 (34.3-49.8)	1	1	0.0005
No	1743	532	30.5 (28.4-32.7)	0.50 (0.35-0.72)	0.52 (0.36 - 0.75)	
**Having fever during the last month**						
Yes	435	175	40.2 (35.6-45.0)	1	1	0.0003
No	1475	427	28.9 (26.8-31.5)	0.61 (0.47-0.78)	0.63 (0.49 - 0.81)	
**Age** (**Years**)						
[15–40]	1360	401	29.5 (27.1-32.0)	1	1	0.0346
[40–55]	550	201	36.5 (32.5-40.7)	1.42 (1.14-1.77)	1.28 (1.02 - 1.61)	
**Distance to rivers** (**Km**)						
> 1.5 Km	1279	364	28.5 (26.0-31.0)	1	1	0.0671
≤ 1.5 Km	631	238	37.7 (33.9-41.6)	1.48 (0.99-2.21)	1.44 (0.97 - 2.14)	

N = number; P = seropositivity to *P*. *falciparum*; RE = Random effect; cOR = Crude Odd ratio; aOR = Adjusted Odd ratio; CI95% = Confident interval 95%.

In contrast, only one risk factor for seropositivity for *P*. *falciparum* was identified: older age (40 years to 55 years). There was a non-significant association of living near lakes and rivers (≤ 1.5 Km) with a higher risk of seropositivity.

### *Plasmodium vivax* seroprevalence

By taking into account the reactivity against PvMSP1_42_ or PvMSP1_19_, the global seropositivity rate to *P*. *vivax* was 17.5% (334/1910 Djiboutian people), CI95% (15.8-19.3). Considering the potential determinants of the *P*. *vivax* malaria infection, according to the results of the multivariate logistic regression analysis (Table 2), some factors were significantly and independently statistically associated with a lower risk of being seropositive for *P*. *vivax*:

– The gender (female gender).

– Never having visited a malaria-endemic country for more than one year.

**Table 2:** **Multivariate logistic regression analysis of *P*. *vivax *infection’s seroprevalence**

	**N**	**P**	**% (95%CI)**	**RE**		
				**cOR (CI95%)**	**aOR (CI95%)**	**p-value**
**Gender**						
Male	742	147	19.8 (17.0-22.9)	1	1	
Female	1168	187	16.0 (14.0-18.2)	0.75 (0.59-0.97)	0.72 (0.56-0.93)	0.0106
**Schooling**						
Schooled	666	97	14.6 (10.6-15.9)	1	1	
Never schooled	1244	237	19.1 (16.9-21.3)	1.32 (1.01-1.73)	1.40 (1.06-1.85)	0.0163
**Staying in a malaria endemic country more than one year**						
Yes	167	45	26.9 (20.4-34.3)	1	1	
No	1743	289	16.6 (14.9-18.4)	0.51 (0.34-0.75)	0.52 (0.35-0.77)	0.0012

N = number; P = seropositivity to *P*. *vivax*; RE = Random effect; cOR = Crude odd ratio; aOR = Adjusted Odd ratio; CI95% = Confident interval 95%.

Having no schooling was significantly and independently statistically associated with a higher risk of being seropositive for *P*. *vivax* infection.

### Mixed infection seroprevalence

The global seropositivity rate of mixed infection was 10.2% (195/1910 Djiboutian people), CI95% (8.9-11.7).The results of the multivariate logistic regression analysis for predicting a mixed infection (Table 3), i.e., seropositivity to serological markers of both *P*. *falciparum* and *P*. *vivax*, showed that some variables were significantly and independently statistically associated with a lower risk of being seropositive for mixed infection:

– The gender (female gender).

– Never having visited a malaria-endemic country for more than one year.

– Not having had a fever during the last month before the study.

**Table 3:** **Multivariate logistic regression analysis of *P*. *falciparum *and *vivax *mixed infection’s seroprevalence**

	**N**	**P**	**% (95%CI)**	**RE**		
				**cOR (CI95%)**	**aOR (CI95%)**	**p**-**value**
**Type of living area**						
Rural	553	43	7.8 (5.7-10.3)	1	1	0.0477
Urban	1357	152	11.2 (9.6-13.0)	1.52 (0.83-2.78)	1.77 (1.01 - 3.11)	
**Gender**						
Male	742	91	12.3 (10.0-14.8)	1	1	0.0006
Female	1168	104	8.9 (7.3-10.7)	0.65 (0.47-0.90)	0.56 (0.40 - 0.78)	
**Educational level**						
Schooled	666	44	6.6 (4.8-8.8)	1	1	0.004
Never Schooled	1244	151	12.1 (10.4-14.1)	1.81 (1.24-2.63)	2.04 (1.38 - 3.02)	
**Staying in a malaria endemic country more than one year**						
Yes	167	31	18.6 (13.0-25.3)	1	1	0.0012
No	1743	164	9.4 (8.1-10.9)	0.39 (0.24-0.64)	0.45 (0.27 - 0.73)	
**Having fever during the last month**						
Yes	435	65	14.9 (11.7-18.6)	1	1	0.0011
No	1475	130	8.8 (7.4-10.4)	0.54 (0.37-0.77)	0.54 (0.38 - 0.78)	
**Distance to rivers or lakes** (**Km**)						
> 1.5 Km	1210	97	8.0 (6.5-9.7)	1	1	0.0190
≤ 1.5 Km	700	98	14.0 (11.5-16.8)	1.83 (1.07-3.13)	1.83 (1.10 - 3.04)	

N = number; P = seropositivity to *P*. *falciparum* and *P*. *vivax*; RE = Random effect; cOR = Crude Odd ratio; aOR = Adjusted Odd ratio; CI95% = Confident interval 95%.

Some factors were significantly and independently statistically associated with a higher risk of being seropositive for mixed infection:

– The type of living area (urban).

– The educational level (never schooled).

– The distance to rivers or lakes (≤ 1.5 Km).

### Bayesian ordinal multinomial logistic regression for the level of response to *P*. *falciparum* antigens

**Figure 1:**
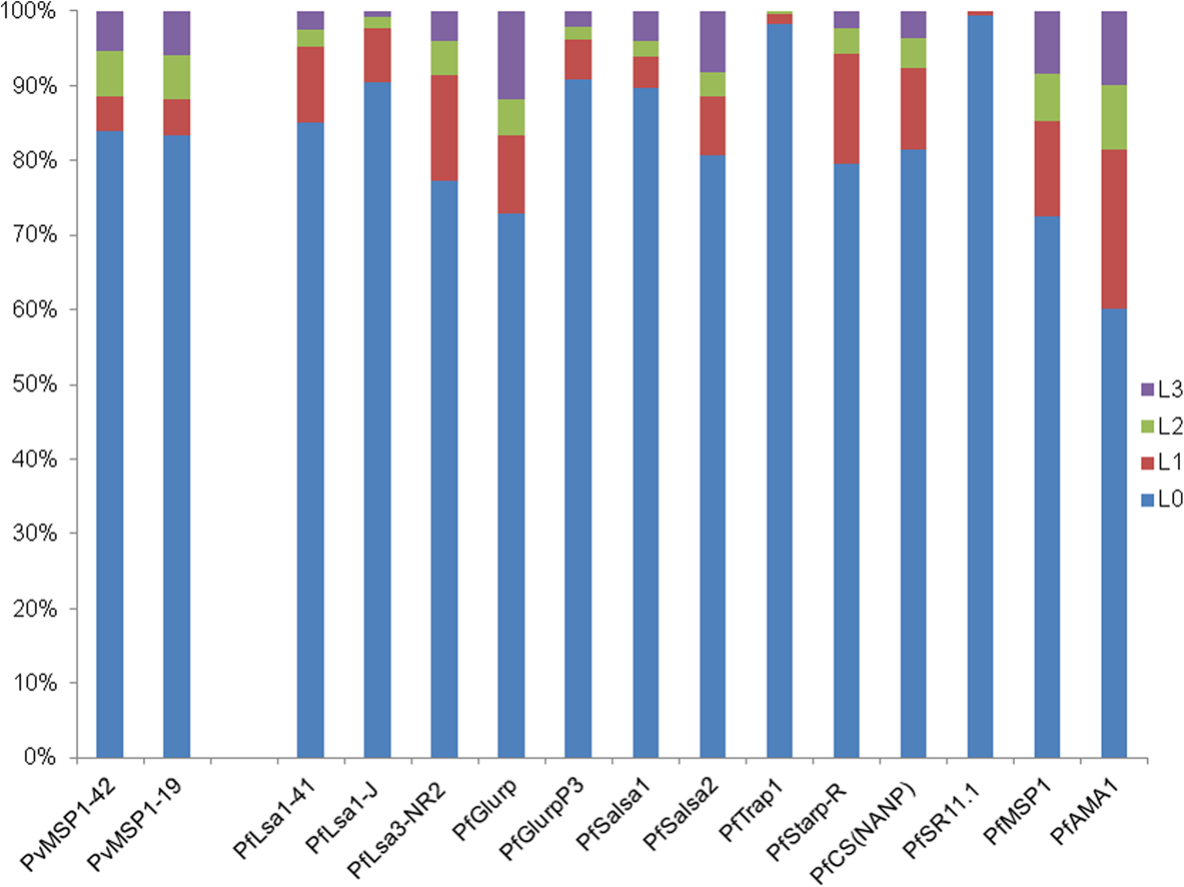
**Proportions of *P***. ***falciparum *and *P***. ***vivax *seropositives to the different peptides and recombinant proteins from the different L**-**groups (for *P. falciparum*, L0 = seronegativity MFI < 1000, L1 = 1000 ≤ MFI < 3800, L2 = 3800 ≤ MFI < 8000, and L3 = MFI ≥ 8000; for *P***. ***vivax, *L0 = seronegativity MFI < 1000, L1 = 1000 ≤ MFI < 2000, L2 = 2000 ≤ MFI < 10000 and L3 = MFI ≥ 10000).**

**Figure 2:**
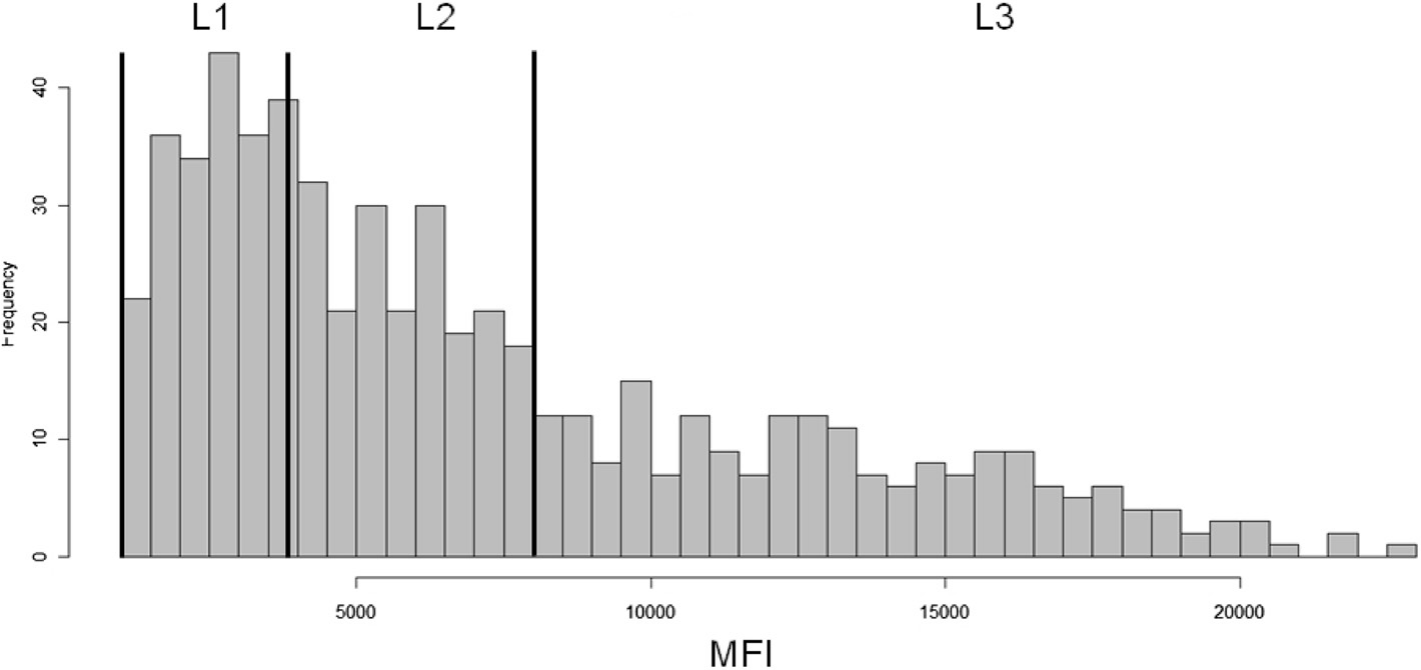
**Distribution of corrected MFI values against the *P***. ***falciparum *antigens in the L1, L2 and L3 groups.**

The proportions of *P*. *falciparum* seropositives to the different peptides and recombinant proteins from the different L-groups (L0 = seronegativity MFI < 1,000, L1 = 1,000 ≤ MFI < 3,800, L2 = 3,800 ≤ MFI < 8,000, and L3 = MFI ≥ 8,000) were illustrated in Figure 1. The distribution of corrected MFI values against the *P*. *falciparum* antigens in the L1, L2 and L3 groups was presented in Figure 2.

According to the results of the Bayesian ordinal multinomial logistic regression analysis (Table 4), some factors were significantly and independently statistically associated with a lower risk of having a high level of immune humeral response to *P*. *falciparum* antigens:

– The gender (female gender).

– The educational level (primary school, secondary, high school or university).

– Never having visited a malaria-endemic country for more than one year.

– Not having had a fever during the last month before the study.

– Rarely to never used bed nets.

**Table 4:** **Multivariate ordinal logistic regression analysis of serological response to *P*. *falciparum *antigens**

	**N**	**P**				**RE**		
		**L1**	**L2**	**L3**	**Total**	**cOR (CI95%)**	**aOR (CI95%)**	**p**-**value**
**Gender**								
Male	742	80	79	97	256	1	1	0.0008
Female	1168	116	127	103	346	0.78 (0.65-0.95)	0.66 (0.54-0.81)	
**Educational level**								
No school	1244	141	145	162	448	1	1	
Primary	410	40	39	27	106	0.61 (0.47-0.77)	0.60 (0.47-0.77)	
Secondary, high school or University	256	15	22	11	48	0.41 (0.30-0.57)	0.39 (0.28-0.55)	
**Staying in a malaria endemic country more than one year**								
Yes	167	19	18	33	70	1	1	0.0019
No	1743	177	188	167	532	0.57 (0.42-0.78)	0.55 (0.40-0.76)	
**Having fever during the last month**								
Yes	435	48	58	69	175	1	1	0.0002
No	1475	148	148	131	427	0.59 (0.47-0.72)	0.59 (0.47-0.73)	
**Distance to rivers and lakes** (**Km**)								
> 1.5 Km	1210	124	121	101	346	1	1	0.0007
≤ 1.5 Km	700	72	85	99	256	1.49 (1.23-1.81)	1.53 (1.25-1.87)	
**Bed nets utilization**								
Often to Always	769	82	90	107	279	1	1	0.0007
Rarely to Never	1141	114	116	93	323	0.67 (0.56-0.81)	0.61 (0.50-0.74)	

N = number; P = seropositivity to *P*. *falciparum*; L = level of intensity of immunological response (seropositivity to *P*. *falciparum* antigens) measured in MFI with 3 levels: 1, 2, 3 corresponding respectively to 1000 ≤ MFI < 3800, 3800 ≤ MFI < 8000, MFI ≥ 8000. The level L0 is not shown here as it’s corresponding to value of MFI < 1000 considered as negative reaction. RE = Random effect; cOR = Crude Odd ratio; aOR = Adjusted Odd ratio; CI95% = Confident interval 95%.

In contrast, only one risk factor was significantly and independently associated statistically with a risk of having a high level of immune humeral response to *P*. *falciparum* antigens:

– The distance to rivers (≤ 1.5 Km).

### Bayesian ordinal multinomial logistic regression for the level of response to *P*. *vivax* antigens

**Figure 3:**
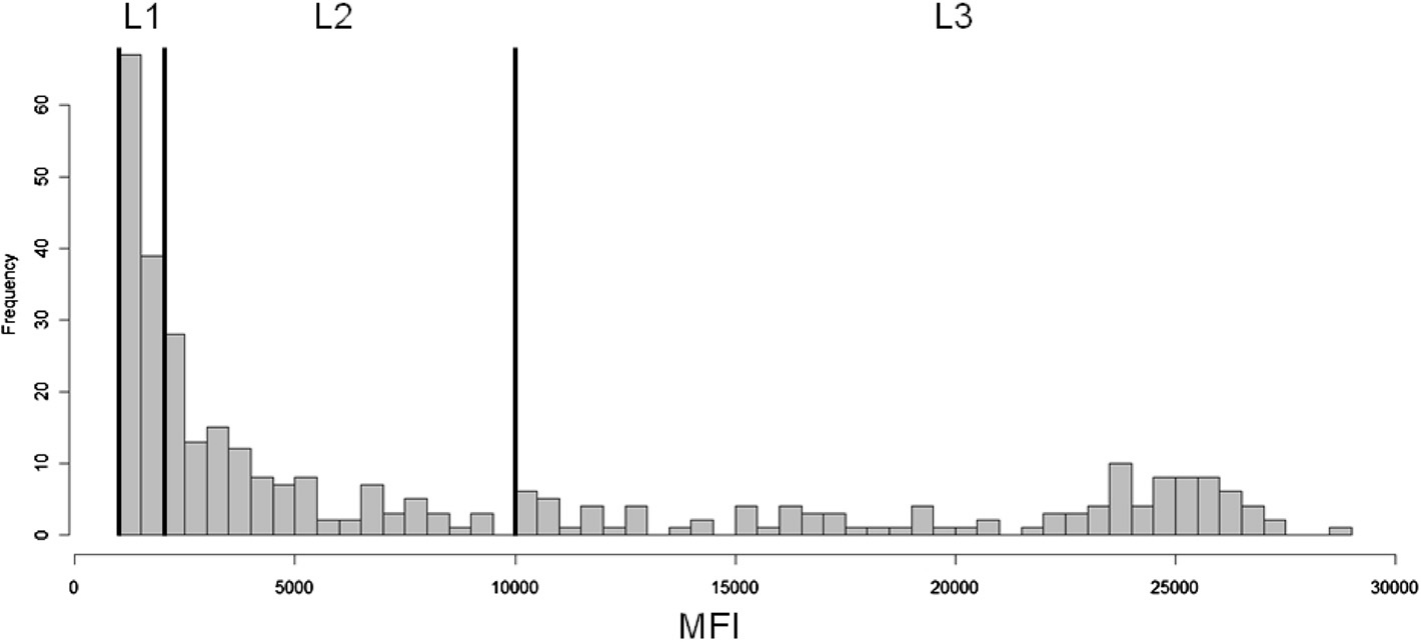
**Distribution of corrected MFI values against the *P. vivax *antigens in the L1, L2 and L3 groups.**

The proportions of *P*. *vivax* seropositives to the different recombinant proteins from the different L-groups (L0 = seronegativity MFI < 1,000, L1 = 1,000 ≤ MFI < 2,000, L2 = 2,000 ≤ MFI < 10,000 and L3 = MFI ≥ 10,000) were illustrated in Figure 1. The distribution of corrected MFI values against the *P*. *vivax* antigens in the L1, L2 and L3 groups was presented in Figure 3. According to the results of the Bayesian ordinal multinomial logistic regression analysis (Table 5), some factors were significantly and independently statistically associated with a lower risk of having a high level of immune humeral response to *P*. *vivax* antigens:

– The gender (female gender).

– Never having visited a malaria-endemic country for more than one year.

– Not having had a fever during the last month before the study.

**Table 5:** **Multivariate ordinal logistic regression analysis of the serological response to *P*. *vivax *antigens**

	**N**	**P**				**RE**		
		**L1**	**L2**	**L3**	**Total**	**cOR (CI95%)**	**aOR (CI95%)**	**p**-**value**
**Gender**								
Male	742	51	52	44	147	1	1	0.0286
Female	1168	54	65	68	187	0.79 (0.62-1.00)	0.74 (0.58-0.94)	
**Educational level**								
Schooled	666	37	35	25	97	1	1	0.0168
Non Schooled	1244	68	82	87	237	1.41 (1.09-1.82)	1.44 (1.11-1.87)	
**Staying in a malaria endemic country more than one year**								
Yes	167	9	18	18	45	1	1	0.0042
No	1743	96	99	94	289	0.52 (0.37-0.75)	0.53 (0.37-0.76)	
**Having fever during the last month**								
Yes	435	25	29	36	90	1	1	0.0447
No	1475	80	88	76	244	0.75 (0.57-0.97)	0.74 (0.56-0.97)	
**Distance to rivers and lakes** (**Km**)								
> 1.5 Km	1210	66	65	60	191	1	1	0.0301
≤ 1.5 Km	700	39	52	52	143	1.39 (1.09-1.76)	1.35 (0.56-0.97)	

N = number; P = seropositivity to *P*. *vivax*; L = level of intensity of immunological response (seropositivity to *P*. *vivax* antigens) measured in MFI with 3 levels: 1, 2, 3 corresponding respectively to 1000 ≤ MFI < 2000, 2000 ≤ MFI < 10000 and MFI ≥ 10000. The level L0 is not shown here as it’s corresponding to value of MFI < 1000 considered as negative reaction. RE = Random effect; cOR = Crude Odd ratio; aOR = Adjusted Odd ratio; CI95% = Confident interval 95%.

Two risk factors were significantly and independently statistically associated with a risk of having a high level of immune humeral response to *P*. *vivax* antigens:

– The distance to rivers (≤ 1.5 Km).

– The educational level (never schooled).

### Geographical repartition

**Figure 4:**
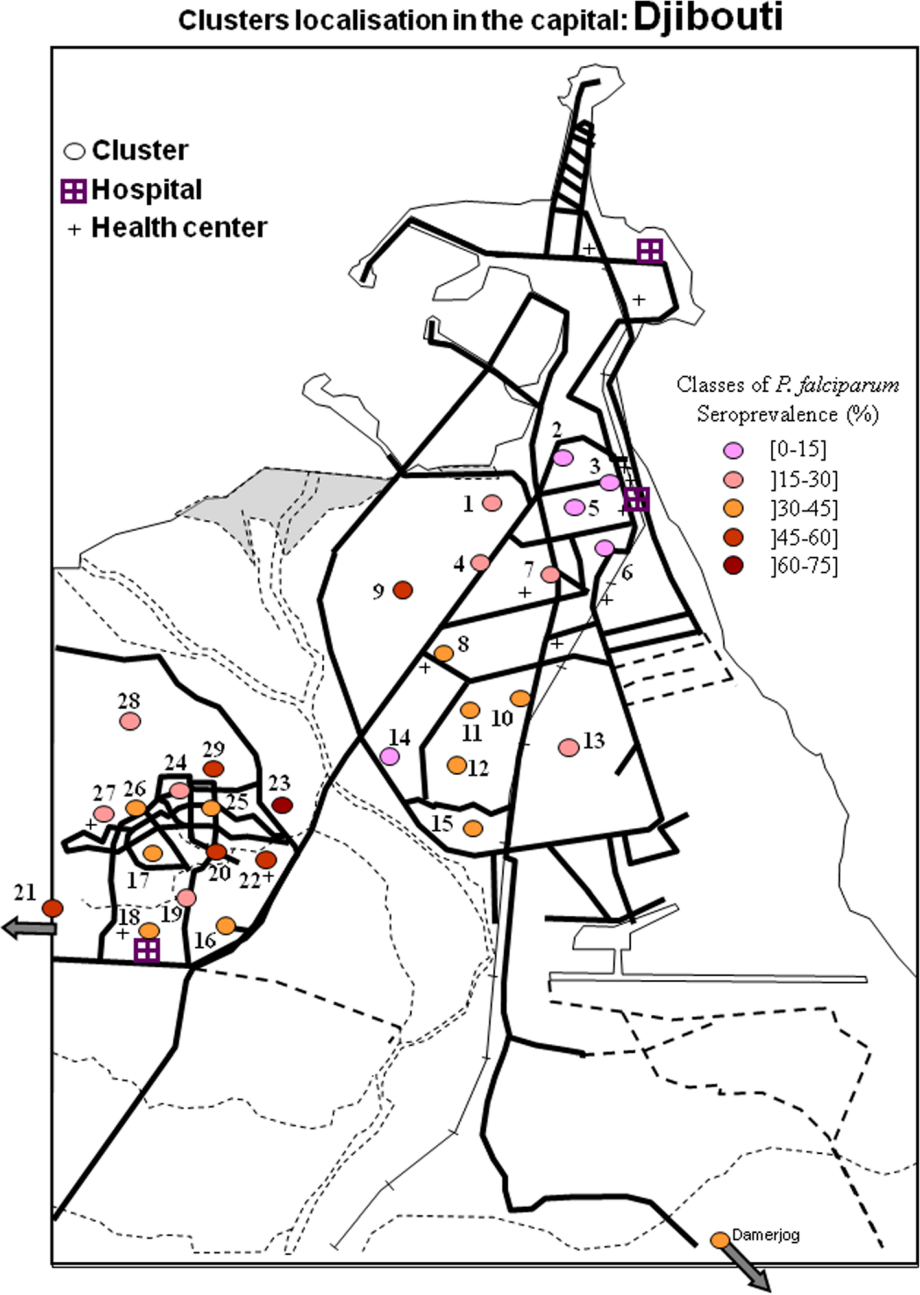
**Map of clusters of *Plasmodium falciparum *seroprevalence in the capital of the republic of Djibouti.**

**Figure 5:**
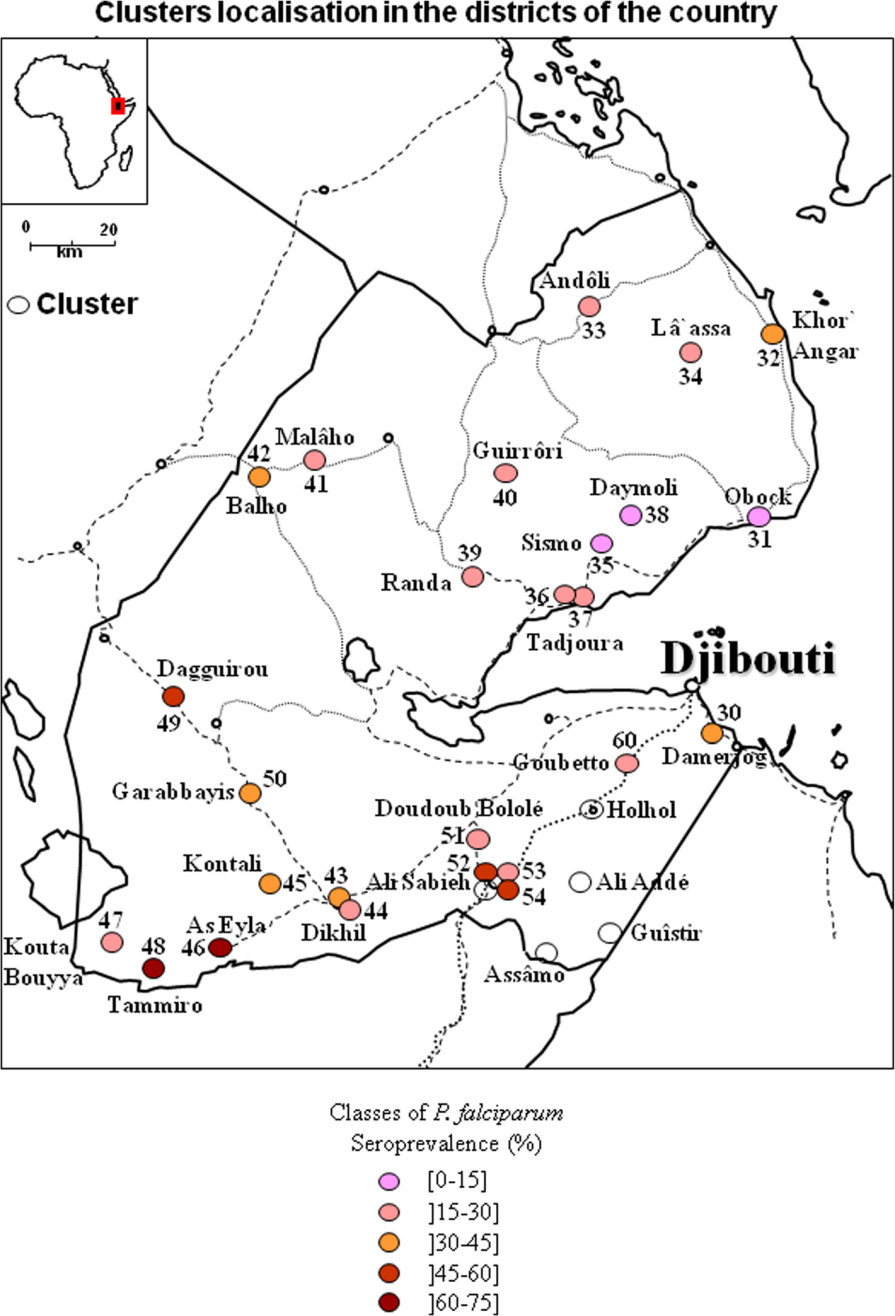
**Map of clusters of *Plasmodium falciparum *seroprevalence in the republic of Djibouti.**

**Figure 6:**
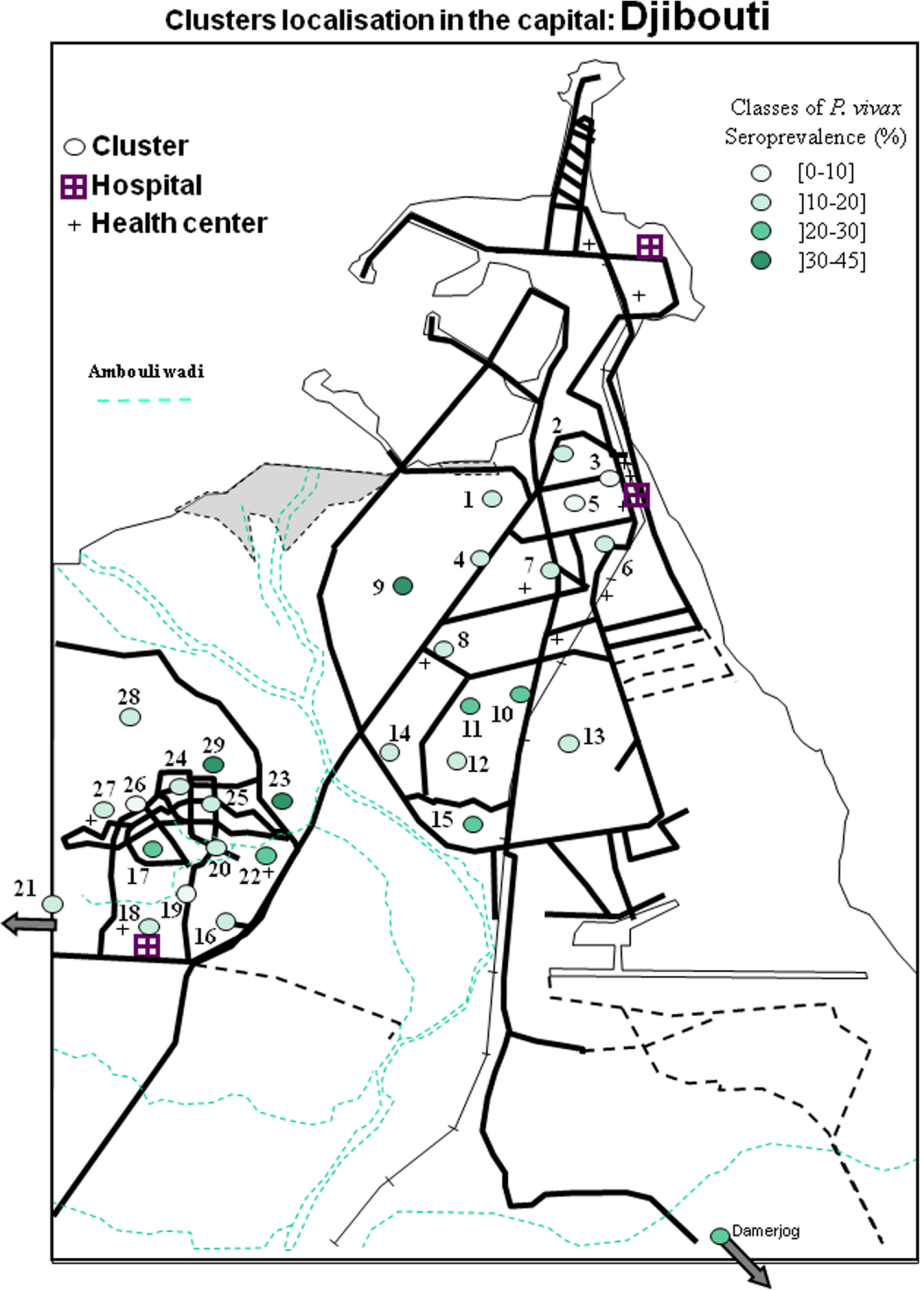
**Map of clusters of *Plasmodium vivax *seroprevalence in the capital of the republic of Djibouti.**

**Figure 7:**
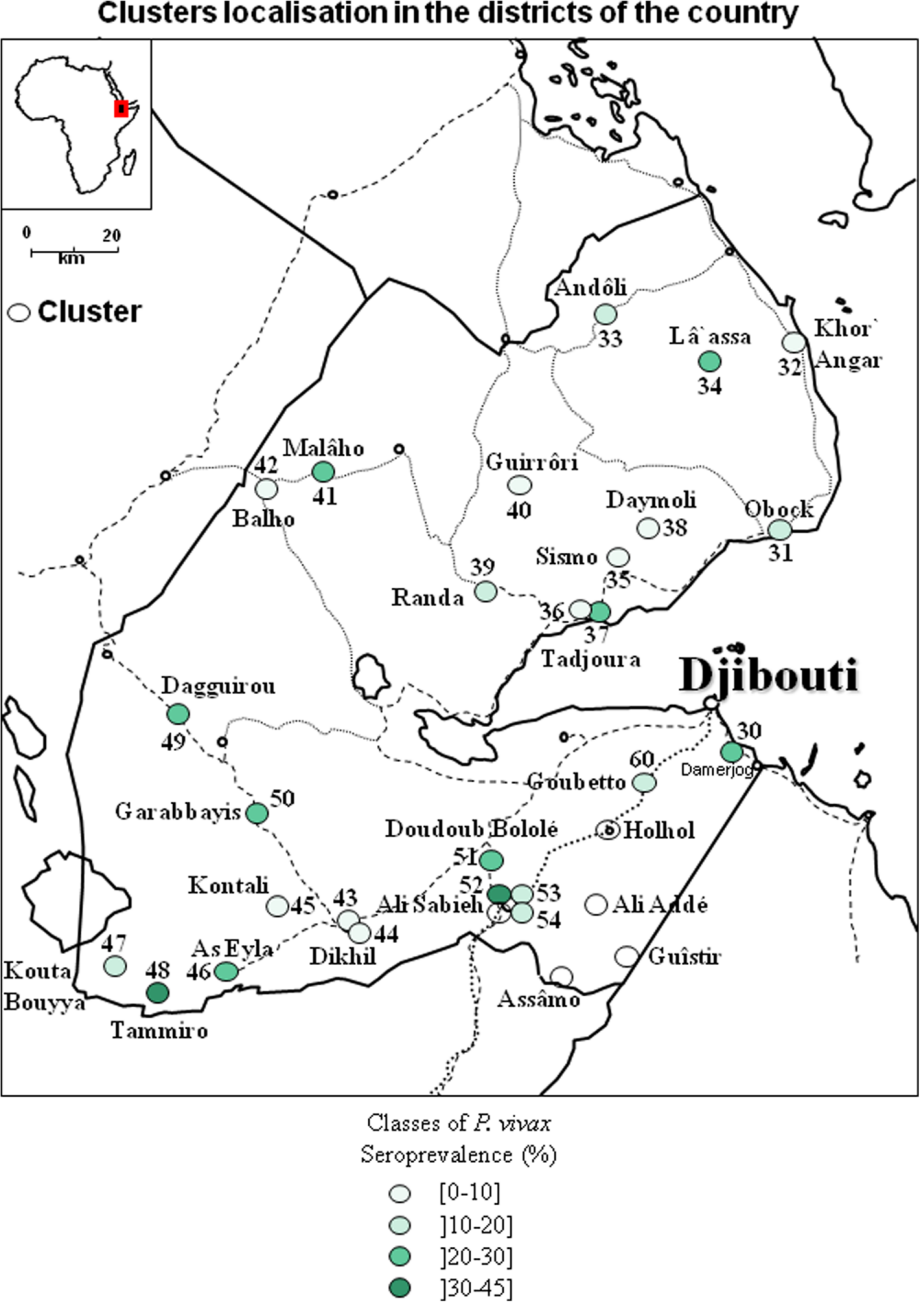
**Map of clusters of *Plasmodium vivax *seroprevalence in the republic of Djibouti.**

The different clusters of *P. falciparum* and *P. vivax* seroprevalence were presented in Figures 4, 5, 6, and 7 and in Additional file 9. For *P*. *falciparum*, the city of Djibouti showed a clustering of low and medium prevalence areas on both sides of the Ambouli wadi. A hotspot was observed in Arhiba, in which more than half of the population (56.4%) (Additional file 10) was seropositive for *P*. *falciparum*. A mean tendency was observed in the upper town (i.e., Quarters 1 to 15), which globally showed a decreasing prevalence when the distance to Ambouli wadi increased. This tendency was also observed in the lower town, i.e., the other side of the Ambouli wadi quarter, with 4 hotspots (Balbala 2, PK12, Balbala 3, and North of Wahle Daba) that had similar prevalence to Arhiba. The most prevalent cluster in the entire country was Balbala 4, in which almost two in three persons (67.5%) were seropositive for *P*. *falciparum*.

In the rest of the country (Additional file 11), the 2 regions in the north (Tadjourah and Obock) exhibited a low prevalence, except for Balho, in which more than one in three persons were seropositive for *P*. *falciparum*. The situation in the South was more concerning, as 3 seropositivity hotspots were observed in Dagguirou (46.9%), Tammiro (64.7%) and As-Eyla (64.7%) in the Region of Dikhil, and 2 seropositivity hotspots were observed in Ali-Sabieh1 (52%) and Ali-Sabieh 3 (45.5%), the capital city of the Region of Ali-Sabieh.

Considering the prevalence of *P*. *vivax*, in Djibouti city, on both sides of Ambouli wadi, an increase in the distance between quarters and the wadi was associated with a decrease in prevalence rates, as was true for *P*. *falciparum*. In the upper town, the only hotspot was in Arhiba, with a 33.3% seroprevalence. In the lower town, two hotspots were observed in Balbala 4 and north of the Wahle Daba, with 35% and 44.4% seroprevalence, respectively.

Of the northern regions, Obock and Tadjourah, the seroprevalence rates in some localities were 20% to 30%, such as in Malâho, La’Assa (Obock Region) and the regional capital Tadjourah (Tadjourah Region). In southern regions, the seroprevalence rates were between 20% and 30%, such as in Dagguirou, Garabayis (or Gour’abouss) and As-Eyla for the Dikhil region and Doudoub Balaleh for the Ali-Sabieh region. Hotspots were observed in Tammiro, with a 32.4% seroprevalence rate (in the region of Dikhil), and in Ali-Sabieh 1, with a 40% seroprevalence rate (the capital of Ali-Sabieh’s region).

## Discussion

The present study was the first to analyse the *P*. *vivax* seroprevalence rate in the Republic of Djibouti. Supplemental information on the *P*. *falciparum* situation in 2002 was also highlighted.

### Serological tools for *P*. *falciparum* infection

The use of different antigenic peptides was dictated by the fact that in countries where malaria transmission occurs, the results of serology may be ambiguous due to cross reactions with other parasitic infections, such as toxoplasmosis [26]. Thus, by increasing the number of antigens and considering the sera reaction to at least two different plasmodial antigens, this enabled limiting the false positive rates. However, this approach using solely antigenic peptides could lead to an underestimation of the level of transmission; thus, other serologic markers were included, such as the recombinant proteins MSP1 and AMA1, the use of which there is a consensus in the literature [27-29].

In 2009, Noor *et al*. observed a *P*. *falciparum* seroprevalence rate of 14.2% in adults above 50 years, 6.9% in children and an average of 9.9% when they tested the reactivity of 4769 sera to one or both serological markers PfMSP1 and PfAMA1 among Djiboutian population [14]. The same method was applied to the present work and produced a seroprevalence rate of 30.0%. Moreover, when the reaction to at least one marker of the 11 peptides, PfMSP1 and PfAMA1 were combined, the seroprevalence increased to 56.6%; finally, a seroprevalence rate of 33.5% was obtained when considering reactions to at least 2 of the 13 markers. In light of these observations, it can be deduced that the prevalence and, indirectly, the *P*. *falciparum* malaria transmission have declined by at least a factor of three in the past seven years. These results were consistent with the needs and obligations that lead to a pre-elimination goal in which the reduction of transmission is the most important key to pre-elimination [15].

### Multivariate logistic regression and Bayesian ordinal multinomial regression analysis

All obtained models predicting the *P*. *falciparum* or *P*. *vivax* seropositivity status or the level of humeral immune response to *P*. *falciparum* or *P*. *vivax* antigens have shown that female gender, a high educational level and never having visited a malaria-endemic country more than one year were protective. Considering the mixed infection seropositivity status, the multivariate logistic regression model showed a protective effect of living at a distance > 1.5 Km from rivers and lakes, in a rural area and not having had fever during the last month before the cross-sectional study. Only the model that predicted the *P*. *falciparum* seropositivity status showed a protective effect of younger ages between 15 and 40 years. As serological tools reflect the cumulative exposition [28], these observations suggest that the older populations were more exposed and that transmission was thus higher in the past. In the city of Djibouti, educational level is generally correlated with the level of wealth and therefore more accessibility to health facilities and prevention measures [30]. Historically, Ethiopia and Djibouti have maintained very important population exchanges in both directions. Therefore, it is normal to see certain Djiboutian populations settle there for long periods due to the far lower living costs when facing economic or social difficulties in the Republic of Djibouti [31]. Carteron in 1978 and Fox in 1991 have shown that Ethiopia was the most important provider of malaria cases to Djibouti [7,32]. This may explain the observation that living in malaria-endemic country (and especially Ethiopia) for more than one year was a risk factor for being seropositive to *P falciparum* or *P*. *vivax*.

### Geographical distributions of *P*. *falciparum* and *P*. *vivax*

In the city of Djibouti, the seroprevalence rates for both parasite species revealed hotspots on both sides of the main wadi, i.e., Ambouli wadi and the quarters of Arhiba and Balbala 4. Arhiba and Balbala 4 are quarters with significant migrant populations who regularly travel to and/or from Ethiopia [31]. There was an association between the decreasing of seroprevalent clusters to both species and the increase of the cluster distance to Ambouli wadi.

In the rest of the country, *P*. *falciparum* seroprevalence rates were higher in the southern regions (Dikhil and Ali-Sabieh), and in particular, hotspots were found along the land routes to Ethiopia, i.e., Tammiro/As-Eyla and Ali-Sabieh. These roads are regularly used by professional truckers, private users and migrants because they are the only two terrestrial roads to Ethiopia [33].

*Plasmodium vivax* seropositivity status was more balanced throughout the Djiboutian territory, with hotspots in the same locations in the southern regions as for *P*. *falciparum*. Because of the possibility of liver persistence of hypnozoïtes, *P*. *vivax* can spread more widely across the entire country in the case of incomplete treatment. *P*. *vivax* can be found where *P*. *falciparum* is no longer detectable and can sometimes be more prevalent, as could be the case in the neighbouring countries [34]. This situation might explain the high seroprevalence rates recorded in northern regions and suggests the occurrence of local transmission foci when *Anopheles* vectors exist.

### The path to pre-elimination

Pre-elimination is a combination of technical feasibility, i.e., the baseline domestic malaria transmission combined with the importation-related transmission, and operational feasibility, which takes into consideration the country’s government status, health status and information on populations at risk [13].

Compared to one recent work [14], the present serological study indirectly indicates that transmission may have decreased by three-fold in the past seven years for *P*. *falciparum*, even though some hotspots were the same as those found in the Dikhil region. This work, once combined with recent information provided by Noor *et al*. has shown that the Republic of Djibouti is likely on the correct path to pre-elimination with benefits that are threatened by the persistence of hotspots such as those in the Dikhil Region. Finding the same hotspots seven years later constitutes a serious threat to the success of the announced goal. Taking into account technical and operational feasibility, pre-elimination is possible only insofar as efficient control methods are implemented at all administrative and executive levels of authority.

Recommendations emerge from the results of the statistical models. If the educational level of the population cannot be increased, pre-elimination will require increased awareness and health education for at-risk populations through methods that are appropriate to local realities. Although being female seems to be protective, health education should target mothers in particular to increase local knowledge, as has been previously done with HIV [35]. Regional collaborative alert systems are also indispensable because the population is regularly moving to a neighbouring country. To act efficiently on hotspots, the mass distribution of bed nets, mass chemotherapy and chemoprophylaxis for both parasites and indoor residual spraying should be performed, followed by serosurveys and vector monitoring. The vector monitoring system is actually debutant and in progress, although it primarily concerns Djibouti city. Border posts should also see an improvement of their monitoring activities and health controls.

## Conclusions

As seen in this study, the *P*. *falciparum* seroprevalence rate was 25.90% in 2002, further studies with the same population would be required to assess if there was a real decrease in transmission of *P. falciparum* in the Republic of Djibouti since 2002.

This is the first study that assessed the prevalence of *P*. *vivax* in the Republic of Djibouti. It is necessary to improve our knowledge of this pathogen in order to create an effective elimination programme.

The protective effect of female gender, educational level and never having visited a malaria-endemic area for more than one year was observed for both *P*. *falciparum* and *P*. *vivax*. For *P*. *falciparum* along, a protective effect was also observed for not having had a fever the last month, living > 1.5 km away from lakes and rivers and being younger in age.

These findings should encourage authorities to improve efforts toward elimination and to begin the final assault against the few persistent hotspots. However, to assess the real pre-elimination status, the precise level of both *P*. *falciparum* and *P*. *vivax* transmission should be regularly monitored by serological methods or other tools and including children less than five years old.

## Competing interests

The authors declare that they have no competing interests.

## Authors’ contributions

BAK, SB, HB, CR and BP conceived and designed the experiments. BAK, LO, MAK, IHF, HMA, AAA, MKW, SNA, HYD and CR collected the data. HB, AP, BAK, SB, VM and CT contributed to reagents/materials/analysis tools. SB, BAK, HB, MAK, VM and CR analysed the data. BAK, SB, AP, VM, CR and BP wrote the paper. All authors read and approved the final manuscript.

## Consent

Blood samples were collected anonymously in accordance with the recommendations of the Djiboutian Ministry of Health, which also gave ethical approval for the study.

## Supplementary Material

Additional file 1 **Bivariate logistic regression analysis of *P. falciparum *infection’s seroprevalence for socio-economic variables.** Click here for file

Additional file 2 **Bivariate logistic regression analysis of *P. falciparum *infection’s seroprevalence for environmental, health and bed net use variables.** Click here for file

Additional file 3 **Bivariate logistic regression analysis of *P. vivax *infection’s seroprevalence for socio-economic variables.** Click here for file

Additional file 4 **Bivariate logistic regression analysis of *P. vivax *infection’s seroprevalence for environmental, health and bed net use variables.** Click here for file

Additional file 5 **Bivariate logistic regression analysis of *P. falciparum *and *P. vivax *mixed infection’s seroprevalence for socio-economic variables.** Click here for file

Additional file 6 **Bivariate logistic regression analysis of *P. falciparum *and *P. vivax *mixed infection’s seroprevalence for environmental, health and bed net use variables.** Click here for file

Additional file 7 **Bivariate ordinal logistic regression analysis of the serological response to *P. falciparum *antigens.** Click here for file

Additional file 8 **Bivariate ordinal logistic regression analysis of the serological response to *P. vivax *antigens.** Click here for file

Additional file 9 ***P. falciparum*, *P. vivax *seroprevalences and geographical localization of the Djiboutian clusters.** Click here for file

Additional file 10 **Map of clusters of *P. falciparum *and *P. vivax *seroprevalence in the capital of the Republic of Djibouti.** Click here for file

Additional file 11 **Map of clusters of *P. falciparum *and *P. vivax *seroprevalence in the Republic of Djibouti.** Click here for file
