# Supplementary material for: Plasmodium vivax and Plasmodium falciparum infections in the Republic of Djibouti: evaluation of their prevalence and potential determinants
Source: Malar J. 2012 Nov 28;11:395. doi: 10.1186/1475-2875-11-395 (PMC3544601; doi:10.1186/1475-2875-11-395)
Supplement: Additional file 1 — Bivariate logistic regression analysis of P. falciparum infection’s seroprevalence for socio-economic variables. [file 1475-2875-11-395-S1.doc]

Additional data 3. Bivariate logistic regression analysis of *P. falciparum* infection’s seroprevalence for socio economic variables

|  | **N** | **P** | **% (95%CI)** | **cOR (95%CI)** | **p-value** |
| --- | --- | --- | --- | --- | --- |
| **Wealth** |  |  |  |  |  |
| Poor | 1582 | 502 | 31.7 (29.4-34.1) | 1.00 |  |
| Less poor | 328 | 100 | 30.5 (25.6-35.8) | 0.85 (0.50-1.45) | 0.5600 |
| **Sex** |  |  |  |  |  |
| Male | 742 | 256 | 34.5 (31.1-38.0) | 1.00 |  |
| Female | 1168 | 346 | 29.6 (27.0-32.3) | 0.78 (0.63-0.96) | 0.0196 |
| **Age** |  |  |  |  |  |
| [15; 20[ | 299 | 85 | 28.4 (23.4-33.9) | 1.00 |  |
| [20; 25[ | 325 | 96 | 29.5 (24.6-34.8) | 1.13 (0.79-1.63) | 0.4916 |
| [25; 30[ | 277 | 82 | 29.6 (24.3-35.4) | 1.14 (0.78-1.66) | 0.5060 |
| [30; 35[ | 275 | 84 | 30.5 (25.2-36.4) | 1.05 (0.72-1.53) | 0.7951 |
| [35; 40[ | 184 | 54 | 29.3 (22.9-36.5) | 1.07 (0.70-1.64) | 0.7434 |
| [40; 45[ | 197 | 75 | 38.1 (31.3-45.2) | 1.50 (1.01-2.24) | 0.0455 |
| [45; 50[ | 128 | 36 | 28.1 (20.5-36.8) | 1.13 (0.70-1.83) | 0.6117 |
| [50; 55] | 225 | 90 | 40.0 (33.6-46.7) | 1.84 (1.25-2.69) | 0.0019 |
| **Schooling** |  |  |  |  |  |
| Schooled | 666 | 148 | 22.2 (19.1-25.6) | 1.00 |  |
| Never schooled | 1244 | 454 | 36.5 (33.8-39.2) | 1.81 (1.51-2.42) | 0.0000 |
| **Educational level** |  |  |  |  |  |
| Never schooled | 1244 | 448 | 36.0 (33.3-38.6) | 1.00 |  |
| Primary | 410 | 106 | 25.9 (21.7-30.4) | 0.65 (0.50-0.84) | 0.0014 |
| Secondary, High School, University | 256 | 48 | 18.8 (14.2-24.1) | 0.45 (0.32-0.64) | 0.0000 |
|  |  |  |  |  |  |

N = total; P = seropositivity to *P. falciparum*;

cOR = crude Odd ratio; CI95% = Confident interval 95%
