# Supplementary material for: Plasmodium vivax and Plasmodium falciparum infections in the Republic of Djibouti: evaluation of their prevalence and potential determinants
Source: Malar J. 2012 Nov 28;11:395. doi: 10.1186/1475-2875-11-395 (PMC3544601; doi:10.1186/1475-2875-11-395)
Supplement: Additional file 2 — Bivariate logistic regression analysis of P. falciparum infection’s seroprevalence for environmental, health and bed net use variables. [file 1475-2875-11-395-S2.doc]

Additional data 4. Bivariate logistic regression analysis of *P. falciparum* infection’s seroprevalence for environmental, health and bednets use variables

|  | **N** | **P** | **% (95%CI)** | **cOR (95%CI)** | **p-value** |
| --- | --- | --- | --- | --- | --- |
| **Geographic zone** |  |  |  |  |  |
| Djibouti-city | 1131 | 370 | 32.7 (30.0-35.3) | 1.00 |  |
| Rest of the country | 779 | 232 | 29.8 (26.6-33.1) | 0.91 (0.62-1.35) | 0.6470 |
| **Urbanism** |  |  |  |  |  |
| Rural | 553 | 174 | 31.5 (27.6-35.5) | 1.00 |  |
| Urban | 1357 | 428 | 31.5 (29.1-34.1) | 0.97 (0.63-1.48) | 0.8800 |
| **Distance to rivers 0** |  |  |  |  |  |
| > 1 km | 1612 | 491 | 30.5 (28.2-32.8) | 1.00 |  |
| ≤ 1 km | 298 | 111 | 37.2 (31.7-43.0) | 1.33 (0.77-2.28) | 0.3010 |
| **Distance to rivers 1** |  |  |  |  |  |
| > 1.5 km | 1279 | 364 | 28.5 (26.0-31.0) | 1.00 |  |
| ≤ 1.5 km | 631 | 238 | 37.7 (33.9-41.6) | 1.48 (0.99-2.21) | 0.0558 |
| **Distance to rivers and lakes 0** |  |  |  |  |  |
| > 1km | 1508 | 460 | 30.5 (28.2-32.9) | 1.00 |  |
| ≤ 1km | 402 | 142 | 35.3 (30.7-40.2) | 1.23 (0.76-1.99) | 0.3980 |
| **Distance to rivers and lakes 1** |  |  |  |  |  |
| > 1.5km | 1210 | 346 | 28.6 (26.1-31.2) | 1.00 |  |
| ≤ 1.5km | 700 | 256 | 36.6 (33.0-40.3) | 1.40 (0.94-2.08) | 0.0963 |
| **Staying abroad in malaria endemic region**  **more than one year** |  |  |  |  |  |
| Yes | 167 | 70 | 41.9 (34.3-49.8) | 1.00 |  |
| No | 1743 | 532 | 30.5 (28.4-32.7) | 0.50 (0.35-0.72) | 0.0002 |
| **Having fever during the last month** |  |  |  |  |  |
| Yes | 435 | 175 | 40.2 (35.6-45.0) | 1.00 |  |
| No | 1475 | 427 | 28.9 (26.8-31.5) | 0,61 (0.47-0.78) | 0.0001 |
| **Utilization of bednets** |  |  |  |  |  |
| Often to Always | 769 | 279 | 36.3 (32.9-39.8) | 1.00 |  |
| Rarely to Never | 1141 | 323 | 28.3 (25.7-31.0) | 0.90 (0.69-1.19) | 0.4740 |
|  |  |  |  |  |  |

N = total; P = seropositivity to *P. falciparum*;

cOR = crude Odd ratio; CI95% = Confident interval 95%
