# Supplementary material for: Plasmodium vivax and Plasmodium falciparum infections in the Republic of Djibouti: evaluation of their prevalence and potential determinants
Source: Malar J. 2012 Nov 28;11:395. doi: 10.1186/1475-2875-11-395 (PMC3544601; doi:10.1186/1475-2875-11-395)
Supplement: Additional file 3 — Bivariate logistic regression analysis of P. vivax infection’s seroprevalence for socio-economic variables. [file 1475-2875-11-395-S3.doc]

Additional data 5. Bivariate logistic regression analysis of *P. vivax* infection’s seroprevalence for socio economic variables

|  | **N** | **P** | **% (95%CI)** | **cOR (95%CI)** | **p-value** |
| --- | --- | --- | --- | --- | --- |
| **Wealth** |  |  |  |  |  |
| Poor | 1582 | 276 | 17.4 (15.6-19.4) | 1.00 |  |
| Less poor | 328 | 58 | 17.7 (13.7-22.3) | 0.99 (0.63-1.55) | 0.9170 |
| **Sex** |  |  |  |  |  |
| Male | 742 | 147 | 19.8 (17.0-22.9) | 1.00 |  |
| Female | 1168 | 187 | 16.0 (14.0-18.2) | 0.75 (0.59-0.97) | 0.0259 |
| **Age** |  |  |  |  |  |
| [15; 20[ | 299 | 52 | 17.4 (13.3-22.2) | 1.00 |  |
| [20; 25[ | 325 | 51 | 15.7 (11.9-20.1) | 0.90 (0.58-1.38) | 0.6230 |
| [25; 30[ | 277 | 49 | 17.7 (13.4-22.7) | 1.00 (0.64-1.55) | 0.9860 |
| [30; 35[ | 275 | 55 | 20.0 (15.4-25.1) | 1.15 (0.75-1.76) | 0.5320 |
| [35; 40[ | 184 | 34 | 18.5 (13.1-24.9) | 1.06 (0.65-1.73) | 0.8100 |
| [40; 45[ | 197 | 33 | 16.8 (11.8-22.7) | 0.87 (0.53-1.43) | 0.5910 |
| [45; 50[ | 128 | 21 | 16.4 (10.5-24.0) | 0.96 (0.54-1.69) | 0.8840 |
| [50; 55] | 225 | 40 | 17.8 (13.0-23.4) | 1.05 (0.66-1.67) | 0.8330 |
| **Schooling** |  |  |  |  |  |
| Schooled | 666 | 97 | 14.6 (10.6-15.9) | 1.00 |  |
| Never schooled | 1244 | 237 | 19.1 (16.9-21.3) | 1.32 (1.01-1.73) | 0.0441 |
| **Educational level** |  |  |  |  |  |
| Never schooled | 1244 | 235 | 18.9 (16.8-21.2) | 1.00 |  |
| Primary | 410 | 63 | 15.4 (12.0-19.2) | 0.81 (0.59-1.12) | 0.2020 |
| Secondary, High School, University | 256 | 36 | 14.1 (10.0-18.9) | 0.75 (0.50-1.11) | 0.1500 |
|  |  |  |  |  |  |

N = total; P = seropositivity to *P. vivax*;

cOR = crude Odd ratio; CI95% = Confident interval 95%
