# Supplementary material for: Plasmodium vivax and Plasmodium falciparum infections in the Republic of Djibouti: evaluation of their prevalence and potential determinants
Source: Malar J. 2012 Nov 28;11:395. doi: 10.1186/1475-2875-11-395 (PMC3544601; doi:10.1186/1475-2875-11-395)
Supplement: Additional file 4 — Bivariate logistic regression analysis of P. vivax infection’s seroprevalence for environmental, health and bed net use variables. [file 1475-2875-11-395-S4.doc]

Additional data 6.Bivariate logistic regression analysis of *P. vivax* infection’s seroprevalence for environmental, health and bednets use variables

|  | **N** | **P** | **% (95%CI)** | **cOR (95%IC)** | **p-value** |
| --- | --- | --- | --- | --- | --- |
| **Geographic zone** |  |  |  |  |  |
| Djibouti-city | 1131 | 204 | 18.0 (15.8-20.4) | 1.00 |  |
| Rest of the country | 779 | 130 | 16.7 (14.1-19.5) | 0.91 (0.65-1.28) | 0.5870 |
| **Urbanism** |  |  |  |  |  |
| Rural | 553 | 91 | 16.5 (13.5-19.8) | 1.00 |  |
| Urban | 1357 | 243 | 17.9 (15.9-20.1) | 1.11 (0.77-1.60) | 0.5790 |
| **Distance to rivers 0** |  |  |  |  |  |
| > 1km | 1612 | 273 | 16.9 (15.1-18.9) | 1.00 |  |
| ≤ 1km | 298 | 61 | 20.5 (16.0-25.5) | 1.25 (0.79-1.96) | 0.3410 |
| **Distance to rivers 1** |  |  |  |  |  |
| > 1.5km | 1279 | 209 | 16.3 (14.4-18.5) | 1.00 |  |
| ≤ 1.5km | 631 | 125 | 19.8 (16.8-23.1) | 1.22 (0.86-1.73) | 0.2590 |
| **Distance to rivers and lakes 0** |  |  |  |  |  |
| > 1km | 1508 | 252 | 16.7 (14.9-18.7) | 1.00 |  |
| ≤ 1km | 402 | 82 | 20.4 (16.6-24.7) | 1.27 (0.85-1.89) | 0.2450 |
| **Distance to rivers and lakes 1** |  |  |  |  |  |
| > 1.5km | 1210 | 191 | 15.8 (13.8-18.0) | 1.00 |  |
| ≤ 1.5km | 700 | 143 | 20.4 (17.5-23.6) | 1.33 (0.96-1.86) | 0.0893 |
| **Staying abroad in malaria endemic region**  **more than one year** |  |  |  |  |  |
| Yes | 167 | 45 | 26.9 (20.4-34.3) | 1.00 |  |
| No | 1743 | 289 | 16.6 (14.9-18.4) | 0.51 (0.34-0.75) | 0.0007 |
| **Having fever during the last month** |  |  |  |  |  |
| Yes | 435 | 90 | 20.7 (17.0-24.8) | 1.00 |  |
| No | 1475 | 244 | 16.5 (14.7-18.5) | 0.76 (0.57-1.02) | 0.0666 |
| **Utilization of bednets** |  |  |  |  |  |
| Often to Always | 769 | 145 | 18.9 (16.2-21.8) | 1.00 |  |
| Rarely to Never | 1141 | 189 | 16.6 (14.5-18.9) | 0.92 (0.69-1.23) | 0.572 |
|  |  |  |  |  |  |

N = total; P = seropositivity to *P. vivax*;

cOR = crude Odd ratio; CI95% = Confident interval 95%
