# Supplementary material for: Plasmodium vivax and Plasmodium falciparum infections in the Republic of Djibouti: evaluation of their prevalence and potential determinants
Source: Malar J. 2012 Nov 28;11:395. doi: 10.1186/1475-2875-11-395 (PMC3544601; doi:10.1186/1475-2875-11-395)
Supplement: Additional file 5 — Bivariate logistic regression analysis of P. falciparum and P. vivax mixed infection’s seroprevalence for socio-economic variables. [file 1475-2875-11-395-S5.doc]

Additional file 5. Bivariate logistic regression analysis of *P. falciparum* and *P. vivax* mixed infection’s seroprevalence for socio economic variables

|  | **N** | **P** | **% (95%CI)** | **cOR (95%IC)** | **p-value** |
| --- | --- | --- | --- | --- | --- |
| **Wealth** |  |  |  |  |  |
| Poor | 1582 | 154 | 9.7 (8.3-11.3) | 1.00 |  |
| Less poor | 328 | 41 | 12.5 (9.1-16.1) | 1.23 (0.60-2.55) | 0.5720 |
| **Sex** |  |  |  |  |  |
| Male | 742 | 91 | 12.3 (10.0-14.8) | 1.00 |  |
| Female | 1168 | 104 | 8.9 (7.3-10.7) | 0.65 (0.47-0.90) | 0.0094 |
| **Age** |  |  |  |  |  |
| [15; 20[ | 299 | 23 | 7.7 (4.9-11.3) | 1.00 |  |
| [20; 25[ | 325 | 38 | 11.7 (8.4-15.7) | 1.73 (0.98-3.06) | 0.0573 |
| [25; 30[ | 277 | 29 | 10.5 (7.1-14.7) | 1.43 (0.79-2.61) | 0.2418 |
| [30; 35[ | 275 | 34 | 12.4 (8.7-16.8) | 1.63 (0.91-2.92) | 0.0996 |
| [35; 40[ | 184 | 16 | 8.7 (5.1-13.7) | 1.11 (0.55-2.23) | 0.7700 |
| [40; 45[ | 197 | 24 | 12.2 (8.0-17.6) | 1.56 (0.83-2.93) | 0.1694 |
| [45; 50[ | 128 | 9 | 7.0 (3.3-12.9) | 1.00 (0.43-2.29) | 0.9918 |
| [50; 55] | 225 | 22 | 9.8 (6.2-14.4) | 1.41 (0.74-2.67) | 0.2931 |
| **Schooling** |  |  |  |  |  |
| Schooled | 666 | 44 | 6.6 (4.8-8.8) | 1.00 |  |
| Never schooled | 1244 | 151 | 12.1 (10.4-14.1) | 1.81 (1.24-2.63) | 0.0022 |
| **Educational level** |  |  |  |  |  |
| Never schooled | 1244 | 148 | 11.9 (10.2-13.8) | 1.00 |  |
| Primary | 410 | 28 | 6.8 (4.6-9.7) | 0.58 (0.37-0.90) | 0.0182 |
| Secondary, High School, University | 256 | 19 | 7.4 (4.5-11.3) | 0.67 (0.30-0.93) | 0.1445 |
|  |  |  |  |  |  |

N = total; P = seropositivity to *P. falciparum* and *P. vivax*;

cOR = crude Odd ratio; CI95% = Confident interval 95%
