# Supplementary material for: Plasmodium vivax and Plasmodium falciparum infections in the Republic of Djibouti: evaluation of their prevalence and potential determinants
Source: Malar J. 2012 Nov 28;11:395. doi: 10.1186/1475-2875-11-395 (PMC3544601; doi:10.1186/1475-2875-11-395)
Supplement: Additional file 6 — Bivariate logistic regression analysis of P. falciparum and P. vivax mixed infection’s seroprevalence for environmental, health and bed net use variables. [file 1475-2875-11-395-S6.doc]

Additional data 8. Bivariate logistic regression analysis of *P. falciparum* and *P. vivax* mixed infection’s seroprevalence for environmental, health and bednets use variables

|  | **N** | **P** | **% (95%CI)** | **cOR (95%IC)** | **p-value** |
| --- | --- | --- | --- | --- | --- |
| **Geographic zone** |  |  |  |  |  |
| Djibouti-city | 1131 | 130 | 11.5 (9.7-13.5 | 1.00 |  |
| Rest of the country | 779 | 65 | 8.3 (6.5-10.5) | 0.70 (0.40-1.22) | 02050 |
| **Urbanism** |  |  |  |  |  |
| Rural | 553 | 43 | 7.8 (5.7-10.3) | 100 |  |
| Urban | 1357 | 152 | 11.2 (9.6-13.0) | 1.52 (0.83-2.78) | 0.1790 |
| **Distance to rivers 0** |  |  |  |  |  |
| > 1km | 1612 | 148 | 9.2 (7.8-10.7) | 1,00 |  |
| ≤ 1km | 298 | 47 | 15.8 (11.8-20.4) | 1.80 (0.10-3.67) | 0.0900 |
| **Distance to rivers 1** |  |  |  |  |  |
| > 1.5km | 1279 | 104 | 8.1 (6.7-9.8) | 1.00 |  |
| ≤ 1.5km | 631 | 91 | 14.4 (11.8-17.4) | 1.84 (1.06-3.17) | 0.0292 |
| **Distance to rivers and lakes 0** |  |  |  |  |  |
| > 1km | 1508 | 140 | 9.3 (7.9-10.9) | 1.00 |  |
| ≤ 1km | 402 | 55 | 13.7 (10.5-17.4) | 1.54 (0.80-2.95) | 0.1960 |
| **Distance to rivers and lakes 1** |  |  |  |  |  |
| > 1.5km | 1210 | 97 | 8.0 (6.5-9.7) | 1.00 |  |
| ≤ 1.5km | 700 | 98 | 14.0 (11.5-16.8) | 1.83 (1.07-3.13) | 0.0276 |
| **Staying abroad in malaria endemic region**  **more than one year** |  |  |  |  |  |
| Yes | 167 | 31 | 18.6 (13.0-25.3) | 1.00 |  |
| No | 1743 | 164 | 9.4 (8.1-10.9) | 0.39 (0.24-0.64) | 0.0001 |
| **Having fever during last month** |  |  |  |  |  |
| Yes | 435 | 65 | 14.9 (11.7-18.6) | 1.00 |  |
| No | 1475 | 130 | 8.8 (7.4-10.4) | 0.54 (0.37-0.77) | 0.0007 |
| **Utilization of bednets** |  |  |  |  |  |
| Often to Always | 669 | 100 | 13.0 (10.7-15.6) | 1.00 |  |
| Rarely to Never | 1141 | 95 | 8.3 (6.8-10.1) | 0.80 (0.53-1.20) | 0.279 |
|  |  |  |  |  |  |

N = total; P = seropositivity to *P. falciparum* and *P.vivax*;

cOR = crude Odd ratio; CI95% = Confident interval 95%
