# Supplementary material for: Plasmodium vivax and Plasmodium falciparum infections in the Republic of Djibouti: evaluation of their prevalence and potential determinants
Source: Malar J. 2012 Nov 28;11:395. doi: 10.1186/1475-2875-11-395 (PMC3544601; doi:10.1186/1475-2875-11-395)
Supplement: Additional file 7 — Bivariate ordinal logistic regression analysis of the serological response to P. falciparum antigens. [file 1475-2875-11-395-S7.doc]

| Supplementary data 9. Bivariate ordinal logistic regression analysis of serological response to *P. falciparum* antigens | | | | | | | | | | | | | | |
| --- | --- | --- | --- | --- | --- | --- | --- | --- | --- | --- | --- | --- | --- | --- |
|  | **L0**  **MFI < 1000** | |  | **L1**  **1000 ≤ MFI < 3800** | |  | **L2**  **3800 ≤ MFI < 8000** | |  | **L3**  **MFI ≥ 8000** | | **Total** | **cOR (95%CI)** | ***p-value*** |
|  | **N** | **% (95%CI)** |  | **N** | **% (95%CI)** |  | **N** | **% (95%CI)** |  | **N** | **% (95%CI)** | **N** |  |  |
| **Living area** | | | | | | | | | | | | | | |
| **Djibouti-city** | 761 | 67.3 (64.5-70.0) |  | 112 | 9.9 (8.2-11.8) |  | 136 | 12.0 (10.2-14.1) |  | 122 | 10.8 (9.0-12.7) | 1131 | **1.00** |  |
| **Rest of the country** | 547 | 70.2 (66.9-73.4) |  | 84 | 10.8 (8.7-13.2) |  | 70 | 9.0 (7.1-11.2) |  | 78 | 10.0 (8.0-12.3) | 779 | **0.86** (0.71-1.05) | **0.18947** |
| **Total** | 1308 | 68.5 (66.3-70.6) |  | 196 | 10.3 (8.9-11.7) |  | 206 | 10.8 (9.4-12.3) |  | 200 | 10.5 (9.1-11.9) | 1910 |  |  |
|  |  |  |  |  |  |  |  |  |  |  |  |  |  |  |
| **Type of living area** | | | | | | | | | | | | | | |
| **Rural** | 379 | 68.5 (64.5-72.4) |  | 65 | 11.8 (9.2-14.7) |  | 53 | 9.6 (7.3-12.3) |  | 56 | 10.1 (7.7-12.9) | 553 | **1.00** |  |
| **Urban** | 929 | 68.5 (65.9-70.9) |  | 131 | 9.7 (8.1-11.4) |  | 153 | 11.3 (9.6-13.1) |  | 144 | 10.6 (9.0-12.4) | 1357 | **1.03** (0.83-1.27) | **0.80499** |
| **Total** | 1308 | 68.5 (66.3-70.6) |  | 196 | 10.3 (8.9-11.7) |  | 206 | 10.8 (9.4-12.3) |  | 200 | 10.5 (9.1-11.9) | 1910 |  |  |
|  |  |  |  |  |  |  |  |  |  |  |  |  |  |  |
| **Distance to rivers**  **and lakes** | | | | | | | | | | | | | | |
| **> 1.5 Km** | 864 | 71.4 (68.8-73.9) |  | 124 | 10.2 (8.6-12.1) |  | 121 | 10.0 (8.4-11.8) |  | 101 | 8.3 (6.9-10.1) | 1210 | **1.00** |  |
| **≤ 1.5 Km** | 444 | 63.4 (59.7-67.0) |  | 72 | 10.3 (8.1-12.8) |  | 85 | 12.1 (9.8-14.8) |  | 99 | 14.1 (11.6-16.9) | 700 | **1.49** (1.23-1.81) | **0.00654** |
| **Total** | 1308 | 68.5 (66.3-70.6) |  | 196 | 10.3 (8.9-11.7) |  | 206 | 10.8 (9.4-12.3) |  | 200 | 10.5 (9.1-11.9) | 1910 |  |  |
|  |  |  |  |  |  |  |  |  |  |  |  |  |  |  |
| **Staying in malaria**  **endemic country**  **more than one year** | | | | | | | | | | | | | | |
| **Yes** | 97 | 58.1 (50.2-65.7) |  | 19 | 11.4 (7.0-17.2) |  | 18 | 10.8 (6.5-16.5) |  | 33 | 19.8 (14.0-26.6) | 167 | **1.00** |  |
| **No** | 1211 | 69.5 (67.3-71.6) |  | 177 | 10.2 (8.8-11.7) |  | 188 | 10.8 (9.4-12.3) |  | 167 | 9.6 (8.2-11.1) | 1743 | **0.57** (0.42-0.78) | **0.0132** |
| **Total** | 1308 | 68.5 (66.3-70.6) |  | 196 | 10.3 (8.9-11.7) |  | 206 | 10.8 (9.4-12.3) |  | 200 | 10.5 (9.1-11.9) | 1910 |  |  |
|  |  |  |  |  |  |  |  |  |  |  |  |  |  |  |
|  | | | | | | | | | | | | | | |
|  | **L0**  **MFI < 1000** | |  | **L1**  **1000 ≤ MFI < 3800** | |  | **L2**  **3800 ≤ MFI < 8000** | |  | **L3**  **MFI ≥ 8000** | | **Total** | **cOR (95%CI)** | ***p-value*** |
|  | **N** | **% (95%CI)** |  | **N** | **% (95%CI)** |  | **N** | **% (95%CI)** |  | **N** | **% (95%CI)** | **N** |  |  |
|  | | | | | | | | | | | | | | |
| **Having fever during**  **the last month** | | | | | | | | | | | | | | |
| **Yes** | 260 | 59.8 (55.0-64.4) |  | 48 | 11.0 (8.2-14.4) |  | 58 | 13.3 (10.3-16.9) |  | 69 | 15.9 (12.6-19.6) | 435 | **1.00** |  |
| **No** | 1048 | 71.1 (68.7-73.4) |  | 148 | 10.0 (8.5-11.7) |  | 148 | 10.0 (8.5-11.7) |  | 131 | 8.9 (7.5-10.5) | 1475 | **0.59** (0.47-0.72) | **0.0029** |
| **Total** | 1308 | 68.5 (66.3-70.6) |  | 196 | 10.3 (8.9-11.7) |  | 206 | 10.8 (9.4-12.3) |  | 200 | 10.5 (9.1-11.9) | 1910 |  |  |
|  |  |  |  |  |  |  |  |  |  |  |  |  |  |  |
| **Utilization of bednets** | | | | | | | | | | | | | | |
| **Often to always** | 490 | 63.7 (60.2-67.1) |  | 82 | 10.7 (8.6-13.1) |  | 90 | 11.7 (9.5-14.2) |  | 107 | 13.9 (11.5-16.6) | 769 | **1.00** |  |
| **Rarely to never** | 818 | 71.7 (69.0-74.3) |  | 114 | 10.0 (8.3-11.9) |  | 116 | 10.2 (8.5-12.1) |  | 93 | 8.2 (6.6-9.9) | 1141 | **0.67** (0.56-0.81) | **0.007** |
| **Total** | 1308 | 68.5 (66.3-70.6) |  | 196 | 10.3 (8.9-11.7) |  | 206 | 10.8 (9.4-12.3) |  | 200 | 10.5 (9.1-11.9) | 1910 |  |  |
|  |  |  |  |  |  |  |  |  |  |  |  |  |  |  |
| **Wealth** | | | | | | | | | | | | | | |
| **Poor** | 1080 | 68.3 (65.9-70.6) |  | 173 | 10.9 (9.4-12.6) |  | 176 | 11.1 (9.6-12.8) |  | 153 | 9.7 (8.3-11.2) | 1582 | **1.00** |  |
| **Less poor** | 228 | 69.5 (64.2-74.5) |  | 23 | 7.0 (4.5-10.3) |  | 30 | 9.1 (6.3-12.8) |  | 47 | 14.3 (10.7-18.6) | 328 | **1.03** (0.80-1.32) | **0.8455** |
| **Total** | 1308 | 68.5 (66.3-70.6) |  | 196 | 10.3 (8.9-11.7) |  | 206 | 10.8 (9.4-12.3) |  | 200 | 10.5 (9.1-11.9) | 1910 |  |  |
|  |  |  |  |  |  |  |  |  |  |  |  |  |  |  |
| **Sex** | | | | | | | | | | | | | | |
| **Male** | 486 | 65.5 (62.0-68.9) |  | 80 | 10.8 (8.6-13.2) |  | 79 | 10.6 (8.5-13.1) |  | 97 | 13.1 (10.7-15.7) | 742 | **1.00** |  |
| **Female** | 822 | 70.4 (67.7-73.0) |  | 116 | 9.9 (8.3-11.8) |  | 127 | 10.9 (9.1-12.8) |  | 103 | 8.8 (7.2-10.6) | 1168 | **0.78** (0.65-0.95) | **0.0459** |
| **Total** | 1308 | 68.5 (66.3-70.6) |  | 196 | 10.3 (8.9-11.7) |  | 206 | 10.8 (9.4-12.3) |  | 200 | 10.5 (9.1-11.9) | 1910 |  |  |
|  |  |  |  |  |  |  |  |  |  |  |  |  |  |  |
| **Schooling** | | | | | | | | | | | | | | |
| **Schooled** | 518 | 77.8 (74.4-80.9) |  | 56 | 8.4 (6.4-10.8) |  | 58 | 8.7 (6.7-11.1) |  | 34 | 5.1 (3.6-7.1) | 666 | **1.00** |  |
| **Never schooled** | 790 | 63.5 (60.8-66.2) |  | 140 | 11.3 (9.6-13.1) |  | 148 | 11.9 (10.2-13.8) |  | 166 | 13.3 (11.5-15.4) | 1244 | **2.06** (1.67-2.55) | **0.0006** |
| **Total** | 1308 | 68.5 (66.3-70.6) |  | 196 | 10.3 (8.9-11.7) |  | 206 | 10.8 (9.4-12.3) |  | 200 | 10.5 (9.1-11.9) | 1910 |  |  |
|  | **L0**  **MFI < 1000** | |  | **L1**  **1000 ≤ MFI < 3800** | |  | **L2**  **3800 ≤ MFI < 8000** | |  | **L3**  **MFI ≥ 8000** | | **Total** | **cOR (95%CI)** | ***p-value*** |
|  | **N** | **% (95%CI)** |  | **N** | **% (95%CI)** |  | **N** | **% (95%CI)** |  | **N** | **% (95%CI)** | **N** |  |  |
|  | | | | | | | | | | | | | | |
| **Educational level** | | | | | | | | | | | | | | |
| **Never schooled** | 796 | 64.0 (61.2-66.7) |  | 141 | 11.3 (9.6-13.2) |  | 145 | 11.7 (9.9-13.6) |  | 162 | 13.0 (11.2-15.0) | 1244 | **1.00** |  |
| **Primary school** | 304 | 74.1 (69.6-78.3) |  | 40 | 9.8 (7.1-13.0) |  | 39 | 9.5 (6.9-12.8) |  | 27 | 6.6 (4.4-9.4) | 410 | **0.61** (0.47-0.77) | **0.0051** |
| **Secondary, High school, University** | 208 | 81.3 (75.9-85.8) |  | 15 | 5.9 (3.3-9.5) |  | 22 | 8.6 (5.5-12.7) |  | 11 | 4.3 (2.2-7.6) | 256 | **0.41** (0.30-0.57) | **0.0011** |
| **Total** | 1308 | 68.5 (66.3-70.6) |  | 196 | 10.3 (8.9-11.7) |  | 206 | 10.8 (9.4-12.3) |  | 200 | 10.5 (9.1-11.9) | 1910 |  |  |
|  |  |  |  |  |  |  |  |  |  |  |  |  |  |  |
| **Age** | | | | | | | | | | | | | | |
| **[15; 20[** | 214 | 71.6 (66.1-76.6) |  | 29 | 9.7 (6.6-13.6) |  | 27 | 9.0 (6.0-12.9) |  | 29 | 9.7 (6.6-13.6) | 299 | **1.00** |  |
| **[20; 25[** | 229 | 70.5 (65.2-75.4) |  | 34 | 10.5 (7.4-14.3) |  | 33 | 10.2 (7.1-14.0) |  | 29 | 8.9 (6.1-12.6) | 325 | **1.03** (0.74-1.45) | **0.8517** |
| **[25; 30[** | 195 | 70.4 (64.7-75.7) |  | 23 | 8.3 (5.3-12.2) |  | 28 | 10.1 (6.8-14.3) |  | 31 | 11.2 (7.7-15.5) | 277 | **1.08** (0.76-1.53) | **0.6761** |
| **[30; 35[** | 191 | 69.5 (63.6-74.8) |  | 29 | 10.5 (7.2-14.8) |  | 27 | 9.8 (6.6-14.0) |  | 28 | 10.2 (6.9-14.4) | 275 | **1.09** (0.77-1.55) | **0.6244** |
| **[35; 40[** | 130 | 70.7 (63.5-77.1) |  | 17 | 9.2 (5.5-14.4) |  | 21 | 11.4 (7.2-16.9) |  | 16 | 8.7 (5.1-13.7) | 184 | **1.03** (0.70-1.53) | **0.8722** |
| **[40; 45[** | 122 | 61.9 (54.8-68.7) |  | 22 | 11.2 (7.1-16.4) |  | 29 | 14.7 (10.1-20.5) |  | 24 | 12.2 (8.0-17.6) | 197 | **1.52** (1.05-2.19) | **0.0465** |
| **[45; 50[** | 92 | 71.9 (63.2-79.5) |  | 16 | 12.5 (7.3-19.5) |  | 12 | 9.4 (4.9-15.8) |  | 8 | 6.3 (2.7-11.9) | 128 | **0.93** (0.59-1.45) | **0.7489** |
| **[50; 55]** | 135 | 60.0 (53.3-66.5) |  | 26 | 11.6 (7.7-16.5) |  | 29 | 12.9 (8.8-18.0) |  | 35 | 15.6 (11.1-21.0) | 225 | **1.69** (1.18-2.40) | **0.0134** |
| **Total** | 1308 | 68.5 (66.3-70.6) |  | 196 | 10.3 (8.9-11.7) |  | 206 | 10.8 (9.4-12.3) |  | 200 | 10.5 (9.1-11.9) | 1910 |  |  |
|  |  |  |  |  |  |  |  |  |  |  |  |  |  |  |
| N = individuals; L1, 2 and 3 = level of intensity of seropositivity response to *P. falciparum* antigens measured in MFI corresponding respectively to 1000 ≤ MFI < 3800, 3800 ≤ MFI < 8000, MFI ≥ 8000 and L = 0 to MFI < 1000 considered as negative reaction. cOR = crude Odd ratio; CI95% = Confident interval 95%. | | | | | | | | | | | | | | |
