# Supplementary material for: Plasmodium vivax and Plasmodium falciparum infections in the Republic of Djibouti: evaluation of their prevalence and potential determinants
Source: Malar J. 2012 Nov 28;11:395. doi: 10.1186/1475-2875-11-395 (PMC3544601; doi:10.1186/1475-2875-11-395)
Supplement: Additional file 8 — Bivariate ordinal logistic regression analysis of the serological response to P. vivax antigens. [file 1475-2875-11-395-S8.doc]

| Supplementary data 10. Bivariate ordinal logistic regression analysis of serological response to *P. vivax* antigens | | | | | | | | | | | | | | |
| --- | --- | --- | --- | --- | --- | --- | --- | --- | --- | --- | --- | --- | --- | --- |
|  | **L0**  **MFI < 1000** | |  | **L1**  **1000 ≤ MFI < 2000** | |  | **L2**  **2000 ≤ MFI < 10000** | |  | **L3**  **MFI ≥ 10000** | | **Total** | **cOR (95%CI)** | ***p-value*** |
|  | **N** | **% (95%CI)** |  | **N** | **% (95%CI)** |  | **N** | **% (95%CI)** |  | **N** | **% (95%CI)** | **N** |  |  |
| **Living area** | | | | | | | | | | | | | | |
| **Djibouti-city** | 927 | 82.0 (79.6-84.2) |  | 62 | 5.5 (4.2-7.0) |  | 79 | 7.0 (5.6-8.6) |  | 63 | 5.6 (4.3-7.1) | 1131 | **1.00** |  |
| **Rest of the country** | 649 | 83.3 (80.5-85.9) |  | 43 | 5.5 (4.0-7.4) |  | 38 | 4.9 (3.5-6.6) |  | 49 | 6.3 (4.7-8.2) | 779 | **0.92** (0.72-1.17) | **0.50556** |
| **Total** | 1576 | 82.5 (80.7-84.2) |  | 105 | 5.5 (4.5-6.6) |  | 117 | 6.1 (5.1-7.3) |  | 112 | 5.9 (4.9-7.0) | 1910 |  |  |
|  |  |  |  |  |  |  |  |  |  |  |  |  |  |  |
| **Type of living area** | | | | | | | | | | | | | | |
| **Rural** | 462 | 83.5 (80.2-86.5) |  | 28 | 5.1 (3.4-7.2) |  | 29 | 5.2 (3.5-7.4) |  | 34 | 6.1 (4.3-8.5) | 553 | **1.00** |  |
| **Urban** | 1114 | 82.1 (79.9-84.1) |  | 77 | 5.7 (4.5-7.0) |  | 88 | 6.5 (5.2-7.9) |  | 78 | 5.7 (4.6-7.1) | 1357 | **1.10** (0.84-1.42) | **0.51755** |
| **Total** | 1576 | 82.5 (80.7-84.2) |  | 105 | 5.5 (4.5-6.6) |  | 117 | 6.1 (5.1-7.3) |  | 112 | 5.9 (4.9-7.0) | 1910 |  |  |
|  |  |  |  |  |  |  |  |  |  |  |  |  |  |  |
| **Distance to rivers**  **and lakes** | | | | | | | | | | | | | | |
| **> 1.5 Km** | 1019 | 84.2 (82.0-86.2) |  | 66 | 5.5 (4.2-6.9) |  | 65 | 5.4 (4.2-6.8) |  | 60 | 5.0 (3.8-6.3) | 1210 | **1.00** |  |
| **≤ 1.5 Km** | 557 | 79.6 (76.4-82.5) |  | 39 | 5.6 (4.0-7.5) |  | 52 | 7.4 (6.0-9.6) |  | 52 | 7.4 (6.0-9.6) | 700 | **1.39** (1.09-1.76) | **0.03577** |
| **Total** | 1576 | 82.5 (80.7-84.2) |  | 105 | 5.5 (4.5-6.6) |  | 117 | 6.1 (5.1-7.3) |  | 112 | 5.9 (4.9-7.0) | 1910 |  |  |
|  |  |  |  |  |  |  |  |  |  |  |  |  |  |  |
| **Staying in malaria**  **endemic country**  **more than one year** | | | | | | | | | | | | | | |
| **Yes** | 122 | 73.1 (65.7-79.6) |  | 9 | 5.4 (2.5-10.0) |  | 18 | 10.8 (6.5-16.5) |  | 18 | 10.8 (6.5-16.5) | 167 | **1.00** |  |
| **No** | 1454 | 83.4 (81.6-85.1) |  | 96 | 5.5 (4.5-6.7) |  | 99 | 5.7 (4.6-6.9) |  | 94 | 5.4 (4.4-6.6) | 1743 | **0.52** (0.37-0.75) | **0.0126** |
| **Total** | 1576 | 82.5 (80.7-84.2) |  | 105 | 5.5 (4.5-6.6) |  | 117 | 6.1 (5.1-7.3) |  | 112 | 5.9 (4.9-7.0) | 1910 |  |  |
|  |  |  |  |  |  |  |  |  |  |  |  |  |  |  |
| **Having fever during**  **the last month** | | | | | | | | | | | | | | |
| **Yes** | 345 | 79.3 (75.2-83.0) |  | 25 | 5.7 (3.8-8.4) |  | 29 | 6.7 (4.5-9.4) |  | 36 | 8.3 (5.9-11.3) | 435 | **1.00** |  |
| **No** | 1231 | 83.5 (81.5-85.3) |  | 80 | 5.4 (4.3-6.7) |  | 88 | 6.0 (4.8-7.3) |  | 76 | 5.2 (4.1-6.4) | 1475 | **0.75** (0.57-0.97) | **0.0761** |
| **Total** | 1576 | 82.5 (80.7-84.2) |  | 105 | 5.5 (4.5-6.6) |  | 117 | 6.1 (5.1-7.3) |  | 112 | 5.9 (4.9-7.0) | 1910 |  |  |
|  |  |  |  |  |  |  |  |  |  |  |  |  |  |  |
| |  | **L0**  **MFI < 1000** | |  | **L1**  **1000 ≤ MFI < 2000** | |  | **L2**  **2000 ≤ MFI < 10000** | |  | **L3**  **MFI ≥ 10000** | | **Total** | **cOR (95%CI)** | ***p-value*** | | --- | --- | --- | --- | --- | --- | --- | --- | --- | --- | --- | --- | --- | --- | --- | |  | **N** | **% (95%CI)** |  | **N** | **% (95%CI)** |  | **N** | **% (95%CI)** |  | **N** | **% (95%CI)** | **N** |  |  | | | | | | | | | | | | | | | |
| **Utilization of bednets** | | | | | | | | | | | | | | |
| **Often to always** | 624 | 81.1 (78.2-83.9) |  | 49 | 6.4 (4.8-8.3) |  | 46 | 6.0 (4.4-7.9) |  | 50 | 6.5 (4.9-8.5) | 769 | **1.00** |  |
| **Rarely to never** | 952 | 83.4 (81.1-85.5) |  | 56 | 4.9 (3.7-6.3) |  | 71 | 6.2 (4.9-7.8) |  | 62 | 5.4 (4.2-6.9) | 1141 | **0.86** (0.68-1.69) | **0.257** |
| **Total** | 1576 | 82.5 (80.7-84.2) |  | 105 | 5.5 (4.5-6.6) |  | 117 | 6.1 (5.1-7.3) |  | 112 | 5.9 (4.9-7.0) | 1910 |  |  |
|  |  |  |  |  |  |  |  |  |  |  |  |  |  |  |
| **Wealth** | | | | | | | | | | | | | | |
| **Poor** | 1306 | 82.6 (80.6-84.4) |  | 89 | 5.6 (4.5-6.9) |  | 96 | 6.1 (4.9-7.4) |  | 91 | 5.8 (4.7-7.0) | 1582 | **1.00** |  |
| **Less poor** | 270 | 82.3 (77.7-86.3) |  | 16 | 4.9 (2.8-7.8) |  | 21 | 6.4 (4.0-9.6) |  | 21 | 6.4 (4.0-9.6) | 328 | **1.03** (0.75-1.40) | **0.8686** |
| **Total** | 1576 | 82.5 (80.7-84.2) |  | 105 | 5.5 (4.5-6.6) |  | 117 | 6.1 (5.1-7.3) |  | 112 | 5.9 (4.9-7.0) | 1910 |  |  |
|  |  |  |  |  |  |  |  |  |  |  |  |  |  |  |
| **Sex** | | | | | | | | | | | | | | |
| **Male** | 595 | 80.2 (77.1-83.0) |  | 51 | 6.9 (5.2-8.9) |  | 52 | 7.0 (5.3-9.1) |  | 44 | 5.9 (4.3-7.9) | 742 | **1.00** |  |
| **Female** | 981 | 84.0 (81.8-86.0) |  | 54 | 4.6 (3.5-6.0) |  | 65 | 5.6 (4.3-7.0) |  | 68 | 5.8 (4.5-7.3) | 1168 | **0.79** (0.62-1.00) | **0.0976** |
| **Total** | 1576 | 82.5 (80.7-84.2) |  | 105 | 5.5 (4.5-6.6) |  | 117 | 6.1 (5.1-7.3) |  | 112 | 5.9 (4.9-7.0) | 1910 |  |  |
|  |  |  |  |  |  |  |  |  |  |  |  |  |  |  |
| **Schooling** | | | | | | | | | | | | | | |
| **Schooled** | 569 | 85.4 (82.5-88.0) |  | 37 | 5.6 (3.9-7.6) |  | 35 | 5.3 (3.7-7.2) |  | 25 | 3.8 (2.4-5.5) | 666 | **1.00** |  |
| **Never schooled** | 1007 | 80.9 (78.7-83.1) |  | 68 | 5.5 (4.3-6.9) |  | 82 | 6.6 (5.3-8.1) |  | 87 | 7.0 (5.6-8.6) | 1244 | **1.41** (1.09-1.82) | **0.0390** |
| **Total** | 1576 | 82.5 (80.7-84.2) |  | 105 | 5.5 (4.5-6.6) |  | 117 | 6.1 (5.1-7.3) |  | 112 | 5.9 (4.9-7.0) | 1910 |  |  |
|  |  |  |  |  |  |  |  |  |  |  |  |  |  |  |
| **Educational level** | | | | | | | | | | | | | | |
| **Never schooled** | 1009 | 81.1 (78.8-83.2) |  | 68 | 5.5 (4.3-6.9) |  | 82 | 6.6 (5.3-8.1) |  | 85 | 6.8 (5.5-8.4) | 1244 | **1.00** |  |
| **Primary school** | 347 | 84.6 (80.8-88.0) |  | 24 | 5.9 (3.8-8.6) |  | 20 | 4.9 (3.0-7.4) |  | 19 | 4.6 (2.8-7.1) | 410 | **0.77** (0.57-1.04) | **0.1292** |
| **Secondary, High school, University** | 220 | 85.9 (81.1-90.0) |  | 13 | 5.1 (2.7-8.5) |  | 15 | 5.9 (3.3-9.5) |  | 8 | 3.1 (1.4-6.1) | 256 | **0.69** (0.47-1.01) | **0.0945** |
| **Total** | 1576 | 82.5 (80.7-84.2) |  | 105 | 5.5 (4.5-6.6) |  | 117 | 6.1 (5.1-7.3) |  | 112 | 5.9 (4.9-7.0) | 1910 |  |  |
|  |  |  |  |  |  |  |  |  |  |  |  |  |  |  |
|  |  |  |  |  |  |  |  |  |  |  |  |  |  |  |
|  |  |  |  |  |  |  |  |  |  |  |  |  |  |  |
|  |  |  |  |  |  |  |  |  |  |  |  |  |  |  |
| |  | **L0**  **MFI < 1000** | |  | **L1**  **1000 ≤ MFI < 2000** | |  | **L2**  **2000 ≤ MFI < 10000** | |  | **L3**  **MFI ≥ 10000** | | **Total** | **cOR (95%CI)** | ***p-value*** | | --- | --- | --- | --- | --- | --- | --- | --- | --- | --- | --- | --- | --- | --- | --- | |  | **N** | **% (95%CI)** |  | **N** | **% (95%CI)** |  | **N** | **% (95%CI)** |  | **N** | **% (95%CI)** | **N** |  |  | | | | | | | | | | | | | | | |
| **Age** | | | | | | | | | | | | | | |
| **[15; 20[** | 247 | 82.6 (77.8-86.7) |  | 23 | 7.7 (4.9-11.3) |  | 19 | 6.4 (3.9-9.7) |  | 10 | 3.3 (1.6-6.1) | 299 | **1.00** |  |
| **[20; 25[** | 274 | 84.3 (79.9-88.1) |  | 12 | 3.7 (1.9-6.4) |  | 14 | 4.3 (2.4-7.1) |  | 25 | 7.7 (5.0-11.1) | 325 | **0.95** (0.63-1.43) | **0.7934** |
| **[25; 30[** | 228 | 82.3 (77.3-86.6) |  | 16 | 5.8 (3.3-9.2) |  | 19 | 6.9 (4.2-10.5) |  | 14 | 5.1 (2.8-8.3) | 277 | **1.05** (0.69-1.59) | **0.8316** |
| **[30; 35[** | 220 | 80.0 (74.8-84.6) |  | 18 | 6.5 (3.9-10.1) |  | 16 | 5.8 (3.4-9.3) |  | 21 | 7.6 (4.8-11.4) | 275 | **1.23** (0.82-1.86) | **0.3371** |
| **[35; 40[** | 150 | 81.5 (75.1-86.9) |  | 11 | 6.0 (3.0-10.4) |  | 14 | 7.6 (4.2-12.4) |  | 9 | 4.9 (2.3-9.1) | 184 | **1.10** (0.69-1.75) | **0.6961** |
| **[40; 45[** | 165 | 83.8 (77.9-88.6) |  | 3 | 1.5 (0.3-4.4) |  | 13 | 6.6 (3.6-11.0) |  | 16 | 8.1 (4.7-12.9) | 197 | **1.01** (0.63-1.61) | **0.9828** |
| **[45; 50[** | 107 | 83.6 (76.0-89.5) |  | 9 | 7.0 (3.3-12.9) |  | 8 | 6.3 (2.7-11.9) |  | 4 | 3.1 (0.9-7.8) | 128 | **0.94** (0.55-1.61) | **0.8138** |
| **[50; 55]** | 185 | 82.2 (76.6-87.0) |  | 13 | 5.8 (3.1-9.7) |  | 14 | 6.2 (3.4-10.2) |  | 13 | 5.8 (3.1-9.7) | 225 | **1.06** (0.68-1.65) | **0.8015** |
| **Total** | 1576 | 82.5 (80.7-84.2) |  | 105 | 5.5 (4.5-6.6) |  | 117 | 6.1 (5.1-7.3) |  | 112 | 5.9 (4.9-7.0) | 1910 |  |  |
|  |  |  |  |  |  |  |  |  |  |  |  |  |  |  |
| N = individuals; L 1, 2 and 3 = level of intensity of seropositivity response to *P. vivax* antigens measured in MFI corresponding respectively to 1000 ≤ MFI < 2000, 2000 ≤ MFI < 10000, MFI ≥ 10000 and L = 0 to MFI < 1000 considered as negative reaction. cOR = crude Odd ratio; CI95% = Confident interval 95%. | | | | | | | | | | | | | | |
