# Supplementary material for: Plasmodium vivax and Plasmodium falciparum infections in the Republic of Djibouti: evaluation of their prevalence and potential determinants
Source: Malar J. 2012 Nov 28;11:395. doi: 10.1186/1475-2875-11-395 (PMC3544601; doi:10.1186/1475-2875-11-395)
Supplement: Additional file 9 — P. falciparum, P. vivax seroprevalences and geographical localization of the Djiboutian clusters. [file 1475-2875-11-395-S9.doc]

*P. falciparum* and *P. vivax* seroprevalences and geographical localization of the Djiboutian clusters

|  |  |  |  | ***P. falciparum*** | | ***P. vivax*** | |
| --- | --- | --- | --- | --- | --- | --- | --- |
| Cluster | **Lat^1^** | **Long^2^** | **N** | **%** | **95%(CI)** | **%** | **95%(CI)** |
| Einguela 1 | 11.351696 | 43.083122 | 29 | 17.2 | 5.9-35.8 | 10.3 | 2.2-27.4 |
| Quartier 1 | 11.352780 | 43.084947 | 29 | 3.4 | 0.1-17.8 | 13.8 | 3.9-31.7 |
| Quartier 3 | 11.351974 | 43.090460 | 40 | 12.5 | 4.2-26.8 | 10.0 | 2.8-23.7 |
| Quartier 4-1 | 11.345529 | 43.082842 | 41 | 22.0 | 10.6-37.6 | 19.5 | 8.8-34.9 |
| Quartier 4-2 | 11.351239 | 43.085254 | 35 | 11.4 | 3.2-26.7 | 2.9 | 0.1-14.9 |
| Quartier 5 | 11.345753 | 43.090078 | 39 | 12.8 | 4.3-27.4 | 15.4 | 5.9-30.5 |
| Quartier 6 Centre | 11.345372 | 43.084140 | 39 | 28.2 | 15.0-44.9 | 15.4 | 5.9-30.5 |
| Quartier 6 Lotissement | 11.342944 | 43.081552 | 38 | 36.8 | 21.8-54.0 | 15.8 | 3.0-31.3 |
| Arhiba | 11.345430 | 43.081059 | 39 | 56.4 | 39.6-72.2 | 33.3 | 19.1-50.2 |
| Quartier 7-1 | 11.341459 | 43.084254 | 42 | 42.9 | 27.7-59.0 | 21.4 | 10.3-36.8 |
| Quartier 7-2 | 11.341603 | 43.081787 | 42 | 33.3 | 19.6-49.5 | 23.8 | 12.1-39.5 |
| Quartier 7 bis | 11.335839 | 43.080812 | 42 | 31.0 | 17.6-47.1 | 16.7 | 7.0-31.4 |
| Gabode1-5, Gelleh Battal, Haramous | 11.335720 | 43.085066 | 38 | 18.4 | 7.7-34.3 | 10.5 | 2.9-24.8 |
| Ambouli | 11.340000 | 43.075709 | 40 | 15.0 | 5.7-29.8 | 12.5 | 4.2-26.8 |
| Djebel | 11.334039 | 43.082483 | 39 | 30.8 | 17.0-47.6 | 23.1 | 11.1-39.3 |
| Nagad | 11.331497 | 43.070584 | 44 | 36.4 | 22.4-52.2 | 11.4 | 3.8-24.6 |
| Ancien Balbala | 11.333744 | 43.061884 | 29 | 34.5 | 17.9-54.3 | 24.1 | 10.3-43.5 |
| Bache à Eau | 11.330720 | 43.062118 | 39 | 38.5 | 23.4-55.4 | 12.8 | 4.3-27.4 |
| Balbala-1 | 11.332321 | 43.063694 | 36 | 25.0 | 12.1-42.2 | 8.3 | 1.8-22.5 |
| Balbala-2 | 11.334906 | 43.070320 | 36 | 50.0 | 32.9-67.1 | 19.4 | 8.2-36.0 |
| PK12 | 11.332132 | 43.045503 | 39 | 51.3 | 34.8-67.6 | 17.9 | 7.5-33.5 |
| Balbala-3 | 11.333247 | 43.071318 | 39 | 48.7 | 32.4-65.2 | 25.6 | 13.0-42.1 |
| Balbala-4 | 11.334604 | 43.072810 | 40 | 67.5 | 50.9-81.4 | 35.0 | 20.6-51.7 |
| Barwaqo (Hayabley) | 11.340585 | 43.063536 | 39 | 28.2 | 15.0-44.9 | 12.8 | 4.3-27.4 |
| Cheik-Moussa | 11.335522 | 43.064194 | 35 | 34.3 | 19.1-52.2 | 14.3 | 4.8-30.3 |
| Hayabley-1 | 11.340489 | 43.061891 | 43 | 30.2 | 17.2-46.1 | 7.0 | 1.5-19.1 |
| Hayabley-2 | 11.340433 | 43.060285 | 33 | 24.2 | 11.1-42.3 | 18.2 | 7.0-35.5 |
| Wahleh-Daba-Nord-1 | 11.341652 | 43.061637 | 40 | 30.0 | 16.6-46.5 | 17.5 | 7.3-32.8 |
| Wahleh-Daba-Nord-2 | 11.341084 | 43.064396 | 36 | 58.3 | 40.8-74.5 | 44.4 | 27.9-61.9 |
| Damerjog | 11.485555 | 43.187222 | 31 | 41.9 | 24.5-60.9 | 29.0 | 14.2-48.0 |
| Obock | 11.963055 | 43.290555 | 37 | 10.8 | 3.0-25.4 | 13.5 | 4.5-28.8 |
| Khor Angar | 12.386666 | 43.334722 | 35 | 37.1 | 21.5-55.1 | 8.6 | 1.8-23.1 |
| Andoli | 12.5 | 42.983333 | 31 | 25.8 | 11.9-44.6 | 19.4 | 7.5-37.5 |
| Laassa | 12.355833 | 43.156388 | 33 | 27.3 | 13.3-45.5 | 27.3 | 13.3-45.5 |
| Sismo | 11.514794 | 42.550294 | 34 | 8.8 | 1.9-23.7 | 5.9 | 0.7-19.7 |
| Tadjourah-1 | 11.472217 | 42.524196 | 29 | 17.2 | 5.9-35.8 | 6.9 | 0.8-22.8 |
| Tadjourah-2 | 11.471242 | 42.530374 | 30 | 23.3 | 9.9-42.9 | 30.0 | 14.7-49.4 |
| Daimoli | 12.241111 | 42.245833 | 35 | 11.4 | 3.2-26.7 | 5.7 | 0.7-19.2 |
| Randa | 11.846944 | 42.660555 | 35 | 25.7 | 12.5-43.3 | 14.3 | 4.8-30.3 |
| Guirrôri | 12.066944 | 42.790833 | 35 | 20.0 | 8.4-36.9 | 8.6 | 1.8-23.1 |
| Malâho | 12.052306 | 42.134493 | 36 | 25.0 | 12.1-42.2 | 25.0 | 12.1-42.2 |
| Balho | 12.058333 | 42.195555 | 33 | 33.3 | 18.0-51.8 | 6.1 | 0.7-20.2 |
| Dikhil-1 | 11.062985 | 42.221214 | 30 | 33.3 | 17.3-52.8 | 10.0 | 2.1-26.6 |
| Dikhil-2 | 11.060881 | 42.223792 | 29 | 20.7 | 8.0-39.7 | 6.9 | 0.8-22.8 |
| Kontali | 11.075869 | 42.162713 | 27 | 37.0 | 19.4-57.6 | 3.7 | 0.1-19.0 |
| As Eyla | 11.003611 | 42.1025 | 28 | 64.3 | 44.1-81.4 | 25.0 | 10.7-44.9 |
| Kouta Bouyya | 11.016111 | 41.961944 | 26 | 26.9 | 11.6-47.8 | 19.2 | 6.6-39.4 |
| Tammiro | 10.976388 | 42.01 | 34 | 64.7 | 46.5-80.3 | 32.4 | 17.4-50.5 |
| Dagguirou | 11.616666 | 41.966666 | 32 | 46.9 | 29.1-65.3 | 25.0 | 11.5-43.4 |
| Garabbayis | 11.3975 | 42.153888 | 26 | 34.6 | 17.2-55.7 | 23.1 | 9.0-43.6 |
| Doudoub Bololé | 11.206666 | 42.661944 | 29 | 44.8 | 26.4-64.3 | 17.2 | 5.9-35.8 |
| Ali Sabieh-1 | 11.091994 | 42.420818 | 25 | 52.0 | 31.3-72.2 | 40.0 | 21.1-61.3 |
| Ali Sabieh-2 | 11.092283 | 42.423081 | 35 | 22.9 | 10.4-40.1 | 17.1 | 6.6-33.7 |
| Ali Sabieh-3 | 11.085594 | 42.423856 | 11 | 45.5 | 16.7-76.6 | 18.2 | 2.3-51.8 |
| Goubétto | 11.423888 | 43.000277 | 44 | 15.9 | 6.6-30.1 | 15.9 | 6.6-30.1 |
| **Total** |  |  | **1910** | **31.5** | **29.4-33.7** | **17.5** | **15.8-19.3** |

^1^ Lat = Latitude, for Einguela 1, 11.351696 means 11°35’16.96" N (North); ^2^Long = Longitude, for Einguela 1, 43.083122 means 43°08’31.22" E (East); N = total number of cluster people; % = *Plasmodium* seroprevalence; CI95% = Confident interval of 95%.
