# Supplementary figures and images for: Plasmodium vivax and Plasmodium falciparum infections in the Republic of Djibouti: evaluation of their prevalence and potential determinants
Source: Malar J. 2012 Nov 28;11:395. doi: 10.1186/1475-2875-11-395 (PMC3544601; doi:10.1186/1475-2875-11-395)

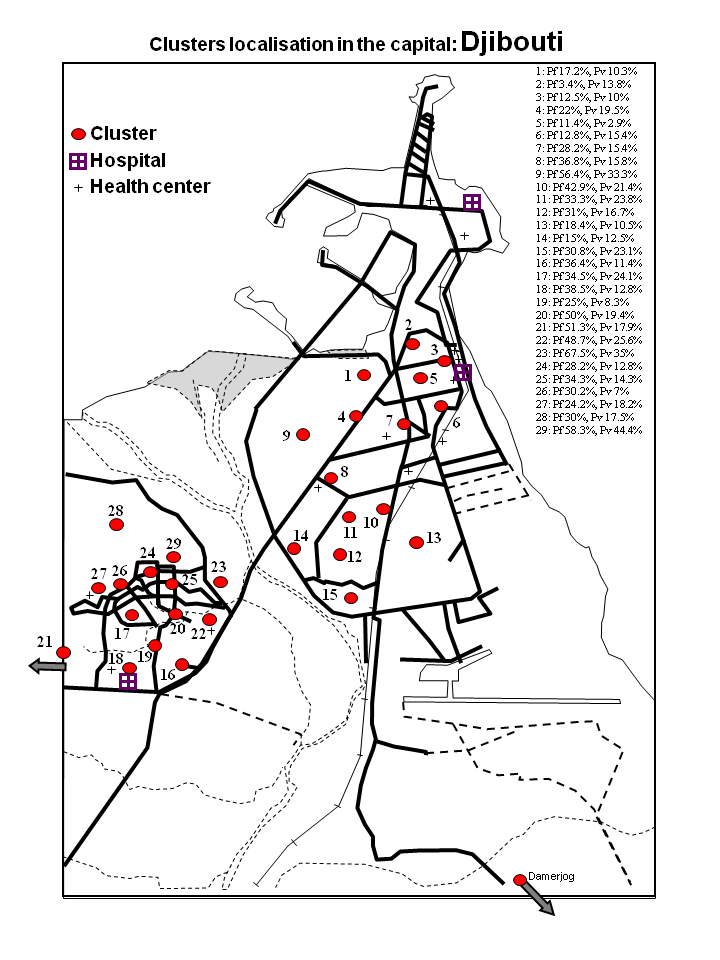

Supplement: Additional file 10 — Map of clusters of P. falciparum and P. vivax seroprevalence in the capital of the Republic of Djibouti. [file 1475-2875-11-395-S10.tiff]

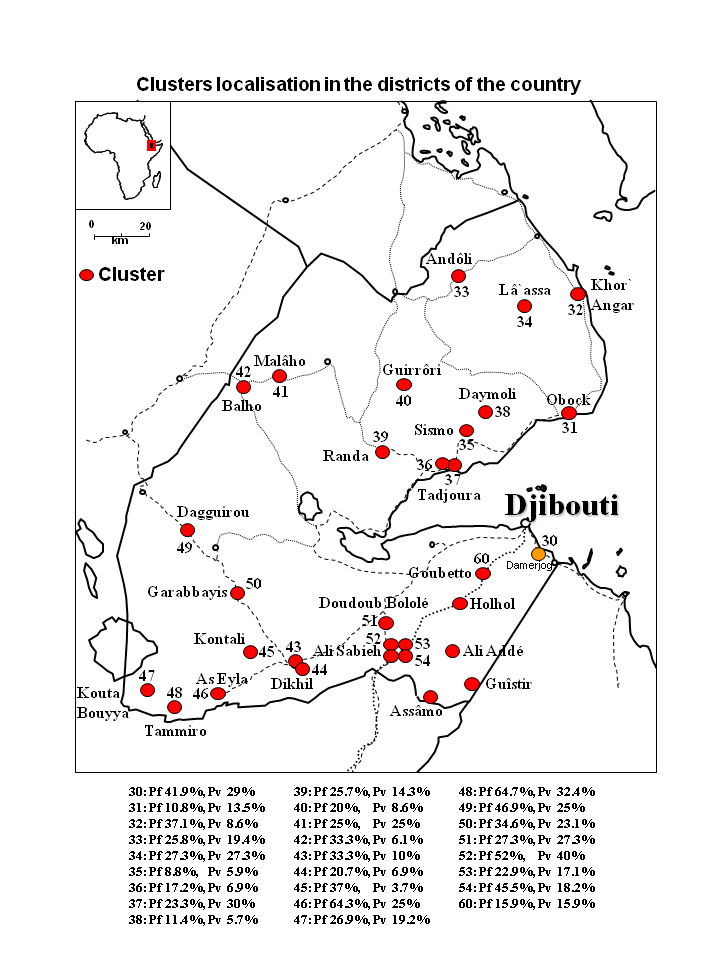

Supplement: Additional file 11 — Map of clusters of P. falciparum and P. vivax seroprevalence in the Republic of Djibouti. [file 1475-2875-11-395-S11.tiff]
